# Human rights violations are associated with forcibly displaced population’s mental health—a systematic review and meta-analysis

**DOI:** 10.3389/fpubh.2024.1454331

**Published:** 2025-01-16

**Authors:** Felix Sisenop, Pallavi Chatarajupalli, Paul A. Bain, Hanna Kaade, Jutta Lindert

**Affiliations:** ^1^Department of Social Work and Health, University of Emden/Leer, Emden, Germany; ^2^Countway Library, Harvard Medical School, Harvard University, Boston, MA, United States; ^3^Department of Palliative Care, Brandenburg Medical School Theodor Fontane (MHB), Brandenburg an der Havel, Germany

**Keywords:** forcibly displaced persons, depression, anxiety, PTSD, human rights violations, global peace index, systematic review, meta-analysis

## Abstract

**Background:**

Little is known about the mental health consequences of human rights violations in forcibly displaced populations. Objective: The objectives of this systematic review are to examine: (1) the prevalence of mental health conditions among forcibly displaced persons; (2) to investigate methodological factors contributing to mental health conditions; and (3) associations between mental health conditions and human rights violations.

**Methods:**

We conducted a systematic review with meta-analyses on the prevalence of anxiety, depression, and posttraumatic stress disorder among forcibly displaced populations and factors contributing to it by searching in databases MEDLINE (Ovid), Embase, Web of Science Core Collection (Clarivate), PsycINFO (EBSCO), Sociological Abstracts (ProQuest), and PTSDPubs (ProQuest). Additionally, we assessed the Global Peace Index. Pooled associations were calculated using a random-effects meta-analysis model. Subgroup analyses were performed for the Global Peace Index, sampling methodology, also we assessed risk of bias.

**Results:**

Of the 8,555 records screened, 55 with *n* = 31,573 participants met the inclusion criteria (*n* = 15,714 males, females, *n* = 15,859 females). Most studies were cross-sectional (*n* = 49). The pooled prevalence rates were 38.90% (95% CI: 29.63; 48.17) for anxiety, 38.16% (95% CI: 32.16; 44.15) for depression and 39.62% (95% CI: 32.87; 46.36) for posttraumatic stress disorder. Analyses by level of human rights violations show anxiety, and depression prevalence rates were higher in countries with very low Global Peace Index than countries with high, moderate and low Global Peace Index (39.84% vs. 16.09%; 41.07% vs. 26.67%). Analyses by risk of bias indicate that the prevalence rate of PTSD was higher in studies with a high risk of bias compared to those with a very high risk of bias (49.27% vs. 29.79%). For anxiety, the prevalence rate was greater with random sampling compared to convenience sampling (44.71% vs. 36.87%). Depression and PTSD prevalence rates were higher with convenience sampling than with random sampling (38.67% vs. 37.70%; 42.83% vs. 35.50%).

**Conclusion:**

Our review suggests that systematic continuous human rights violations are associated with mental health conditions in forcibly displaced persons. To prevent mental health conditions, it is necessary to reduce exposure to human rights violations in the countries forcibly displaced persons come from.

**Systematic review registration:**

https://www.crd.york.ac.uk/prospero/display_record.php?ID=CRD42017076535, PROSPERO registration: CRD42017076535.

## Introduction

Mental health conditions among forcibly displaced populations (FDPs) are a public mental health challenge given the prevalence of mental health conditions and the number of FDPs worldwide (1). Forcibly displaced persons include refugees as defined by the 1951 Refugee Convention (2). We use in this paper the term forcibly displaced persons as an umbrella term which includes refugees and asylum seekers. The number of FDPs was 89.3 million in 2021 (3) and is estimated to be more than 130 million by the end of 2024 (4). A broad range of factors contribute to mental health conditions among FDPs (5, 6). These factors are among other socioeconomic conditions including low household income and poverty (7), food insecurity (8), unemployment and job insecurity (9), financial difficulties (5), violence, conflicts and traumatic events including access to basic human rights such as secure housing and healthcare (10). However, studies suggest, heterogeneous prevalence rates of mental health conditions among FDPs between 1 and 77% for PTSD 4–74% for depression, and anxiety disorders between 2 and 50% (11). Yet, few studies investigated associations between human rights violations and mental health conditions of forcibly displaced persons. This could be because of human rights violations (e.g., restricting the freedom of movement and residence; freedom of thought, expression, religion; and rights to food, health, and livelihood, policies restricting the ability to marry, have children, or travel) have been assessed in few studies (12).

Human rights are fundamental entitlements inherent to every individual, irrespective of factors such as race, gender, nationality, ethnicity, language, or religion. These rights encompass essential freedoms, including the right to life, liberty, freedom from slavery and torture, freedom of expression, and access to education, work and health. They apply universally without any form of discrimination (13). To the best of our knowledge, no systematic review so far investigated the association of human rights violations with FDP’s mental health.

The objectives of this systematic review are therefore to evaluate (1) the prevalence of mental health conditions among FDPs; (2) investigate methodological factors contributing to study results; and (3) evaluate associations between mental health conditions and human rights violations. This study represents a novel contribution to the literature on FDPs mental health. This systematic review is registered with PROSPERO, identifier CRD42017076535.

## Methods

### Search strategy

In this systematic review and meta-analysis, undertaken according to both MOOSE and PRISMA standards, a health-science librarian (PB) developed the search strategy in consultation with the principal investigator (JL). The search included studies on forcibly displaced persons published until June 2022. We searched MEDLINE, Embase, PsycINFO, Scopus, and Web of Science for publications on FDPs and mental health conditions, as defined using a combination of keywords and controlled vocabulary terms applicable to each database in April 2017 and the search was updated in June 2022 (Supplementary Table 1), with no publication type or language restrictions at this stage. Articles with FDPs were identified using MeSH and text keywords for “FDPs,” while “mental health” content in articles was identified using MeSH and text keywords for “mental health” and specifically for “depression,” “anxiety,” and “PTSD.” These terms were adapted for each database. We supplemented the bibliographic database searches by checking the reference lists of identified relevant studies for additional relevant research. We managed the references using ENDNOTE 21.2 through which duplicates were removed. An additional search with a focus on individuals displaced by the war in Ukraine was was performed in the database PubMed in October 2024, however no study provided data on individuals fleeing from the territories occupied by Russia.

### Inclusion and exclusion criteria

Firstly, we developed a study protocol. Based on the protocol, four reviewers (FS, PC, JL and HK) screened titles and abstracts of all potentially eligible publications. We excluded case reports, experimental studies, and studies with no prevalence information on mental health conditions, studies on internally displaced persons, study samples fewer than 100 participants, studies with clinical settings, studies on internally displaced populations as well as studies with populations aged below 18 years. All studies included at this stage were published in English, reported original research using an observational study design (cross-sectional or cohort) and with information on the mental health conditions (anxiety disorders; depressive disorders; trauma and stressor-related disorders). We chose this threshold to enable us to draw on the maximum amount of information in the current literature and to sample broadly to minimize bias. In the second step, following the above criteria, full-text articles were assessed for inclusion by the same reviewers and the same was followed for exclusion of studies. Disagreements between reviewers were solved by consensus (Supplementary Figure 1).

### Data extraction

A standardized data extraction sheet was developed. We extracted (1) study characteristics (e.g., author, year of publication), (2) participants’ characteristics [e.g., age, gender, sample size(s)], (3) study participant’s country by origin and settlement, (4) study design, sampling method (5) measurements (e.g., exposure measure, adjustment/control of confounders, outcome measure for anxiety, depression and PTSD). Three reviewers (FS, PC, HK) extracted and cross-checked data independently for included full-text articles, including study information, participant characteristics, and information needed to calculate pooled estimates of prevalences for each co-occurring mental health condition.

### Assessment of human rights violations

Human rights violations can be assessed in a variety of ways. We used an external dataset, the Global Peace Index (GPI). The GPI uses 23 quantitative and qualitative indicators and measures the status of peace across three domains: the level of societal safety and security, the extent of ongoing domestic and international conflict, and the degree of militarization. These indicators were first selected with the assistance of an expert panel in 2007 and are evaluated by the expert panel on a yearly basis. The scores for each indicator are standardized on a scale of 1–5, whereby qualitative indicators are banded into five groups, while quantitative ones are scored from 1–5 (14). The GPI ranks 172 states and territories (collectively accounting for 99.7 per cent of the world’s population) according to their levels of peacefulness. In the last years, the GPI showed an increase in violence and a decrease in peace (15).

### Data analyses

Prevalence estimates of mental health outcomes are calculated with 95% confidence intervals (CIs) in the pooled data. Random-effects meta-analyses were calculated using the DerSimonian and Laird estimator based on inverse variance weights (16). Heterogeneity was anticipated because of between-study variations in study, design, sampling methods, country of origin, type of exposure and country of settlement. Hence random-effects meta-analysis was used to aggregate the prevalence rates. The heterogeneity among studies was described by I^2^-statistic. I^2^ is not affected by sample size and thus was considered useful for comparisons (17). Additionally, we evaluated potential sources of heterogeneity across studies in three subgroup analyses. First, we investigated the potential effects of human rights violations using the Global Peace Index ranking of countries. The indices of human rights violations were divided into three groups where low, moderate and high GPI countries were compared with very low GPI countries. Studies with samples from more than three countries were excluded from subgroup analysis by GPI in all three outcomes. Further, we analyzed the prevalence rates by risk of bias and sampling method (random and convenience sampling). All analyses were performed using Stata software version 18.5 [Stata Corp].

### Sensitivity analysis

Sensitivity analysis was conducted based on the study quality or risk of bias for 55 studies included. The risk of bias was assessed by two reviewers (FS and PC) using a modified version of quality assessment for quantitative genocide studies (18). This tool evaluates eight domains: ethical approval, external validity and selection bias, misclassification bias, study design, confounders, data collection methods, withdrawals and dropouts, and data analysis. Each domain is rated as strong (1 point), moderate (2 points), or weak (3 points). Based on the cumulative score, studies are categorized into quality ratings: strong, moderate, weak, or very weak. Additionally, the risk of bias was determined based on these quality ratings, with strong-rated studies associated with very low to low risk, moderate-rated studies considered moderate risk, weak-rated studies indicating high risk, and very weak-rated studies reflecting very high risk of bias. We used this tool which is based on the Effective Public Health Practice Project Quality Assessment Tool for Quantitative Studies (EPHPP), as it allows to evaluate the potential risk of bias for populations affected by mass violence and human rights violations (19).

In the subgroup analysis, we considered the level of human rights violations b by human rights violations by GPI score of the FDPs country of origin and study conducted year. We excluded studies that represented samples from more than three countries (20–27), and one study due to lack of GPI score (28). As a result, the number of studies in each subgroup was limited to the number of studies included in the meta-analysis. Therefore, subgroups in all three outcomes were merged when there were three or fewer studies in each category. Further, in anxiety subgroup analysis, study from Carta et al., 2018 (29) was not included due to negative lower bound confidence intervals in the prevalence rates for high, moderate and low GPI subgroups. This is likely because of the large prevalence within the studies in this category. After excluding this study, our analysis yielded a positive confidence interval and presented. In the subgroup analysis by sampling method, the study from Bogic et al., 2012 (28) was not included due to the mixed method sampling method.

## Results

The search yielded 8,555 articles, with 611 articles being added from other sources. After abstract title and abstract screening, 8,314 records were excluded. Another 186 studies were excluded after full-text screening. Overall, 55 studies were included, providing data for 31,573 adult FDPs (Table 1). The additional search on individuals displaced by the war in Ukraine yielded 470 studies. Of those no study fulfilled the inclusion criteria. Supplementary Figure 1 shows the search, inclusion and exclusion process in detail. Characteristics including gender and age distribution, country of origin, study design, sampling strategy, exposure and exposure measures, outcome and outcome measures, confounder and confounder measures as well as prevalence rates of anxiety, depression and PTSD of selected studies are shown in Table 1. 18 of the 55 studies investigated anxiety, 38 depression and 41 PTSD. 49 studies used a cross-sectional study design (20–26, 28–70), three studies a longitudinal study design (27, 71, 72) and one study cohort study design (73) was used.

**Table 1:** Characteristics of included studies.

Author(s), year	Study participants (n, gender (%), age in years)	Refugee country		Study design, sampling methods	Exposure	Exposure measure	Outcomes	Outcome measure	Confounder	Confounder measure	Results: n (prevalence), M (SD)/95% CI
		Origin	Settlement								
Acarturk C et al., 2020^A^ (30); Fuhr DC et al., 2019^B^ (74)	*n* = 1,678 [*n* = 812 males (48%), *n* = 866 females (52%), 18–88]	Syria	Turkey	Cross-sectional, random sampling	War	Own scale	Anxiety, depression, PTSD,	PCL-5, HSCL-25	Age, gender, education, displacement time, living with a chronic disease or disability, lifetime history of mental health treatment	Own scale	Anxiety: *n* = 582* (34.7%), 95% CI: 32.4–37.0, depression: *n* = 606* (36.1%), 95% CI: 33.8–38.4, PTSD: *n* = 329* (19.6%), 95% CI: 17.7–21.5
Ahmad F et al., 2021 (71)	*n* = 1924 [*n* = 937 males (48.86%), *n* = 984 females (51.14%), ≥18]	Syria	Canada	Longitudinal, snowball sampling	Refugee resettlement process	MSPSS, perceived control scale	Depression	PHQ-9	Age, gender, marital status, education, employment status, religion, financial hardship	Own scale	Depression: *n* = 292* (15.2%), 95% CI: 0.137–0.169
Ainamani HE et al., 2020 (31)	*n* = 325 [*n* = 143, males (44%), *n* = 182 females (56%), 18–65]	Democratic Republic of Congo	Uganda	Cross-sectional, convenience sampling	War violence	War-related traumatic events Checklist-25	PTSD	DSM-IV	Gender	Own scale	PTSD: *n* = 285* (87.7%), males *n* = 118 (83.7%), females *n* = 167 (93.8%)
Alpak G et al., 2015 (32)	*n* = 352 [*n* = 179 males (50.9%), *n* = 173 females (49.1%), 18–65]	Syria	Turkey	Cross-sectional, random sampling	Traumatic incidents	Stressful life events screening questionnaire, adapted	PTSD	Diagnostic psychiatric interview, DSM-IV-TR	Age, gender, marital status, number of children, number of people who live together, education, employment status, duration of asylum, health behavior, personal history of medical disorder, family history of psychiatric disorder	Own scales	PTSD: *n* = 118 (33.5%), males: *n* = 38 (32.2%), females: *n* = 80 (67.8%)
Bapolisi AM et al., 2020 (20)	*n* = 387 [*n* = 168 males (43.44%), *n* = 219 females (56.56%), ≥18]	>3 countries^1^	Uganda	Cross-sectional, stratified quota random sampling	Traumatic events in the home country or during displacement	–	Anxiety, depression, PTSD	MINI 7	Perception of stress	Own scale	Anxiety: *n* = 283* (73%), depression: *n* = 224* (58%), PTSD: *n* = 259* (67%)
Basheti IA et al., 2019 (33)	*n* = 186 [*n* = 99 males (53.2%), *n* = 87 females (46.8%), ≥18]	Syria	Jordan	Cross-sectional, convenience sampling	War trauma	HTQ	PTSD	HTQ-16	Age, gender, marital status, education, residential status, smoking, watching TV	Own scale	PTSD: *n* = 72*(38.7%), males M = 2.42 (SD = 0.50), females M = 2.26 (SD = 0.57)
Beiser M et al., 2011 (34)	*n* = 1,603 [*n* = 866 males (54%), *n* = 737 females (46%), ≥18]	Sri Lanka	Canada	Cross-sectional, convenience sampling	Stresses of passage, post migration stress	Own scales	PTSD	WHO-CIDI	Age, gender, marital status, education, employment status, household income below the poverty line, duration of stay in host country	Own scales	PTSD: *n* = 192 (12%)
Berhe SM et al., 2021 (35)	*n* = 786 [*n* = 495 males (63%), *n* = 291 females (37%), 25–45]	Eritrea	Ethiopia	Cross-sectional, random sampling followed by systematic sampling	War trauma	HTQ	Depression	PHQ-9	Age, gender, education, employment status, social support, displacement history, personal history of psychiatric disorder, family history of psychiatric disorder, duration of stay in refugee camp, current presence of family	Own scale, OSSS-3	Depression: *n* = 297* (37.8%), 95% CI: 34.2–41.2
Berthold SM et al., 2014 (36)	*n* = 136 [*n* = 53 males (39%), *n* = 83 females, 32–85]	Cambodia	USA	Cross-sectional, snowball sampling	War	Own scale	Depression, PTSD	HTQ, HSCL	Age, gender, personal history of physical and mental disorders	Own scale	Depression: *n* = 5 (3.7%), PTSD: *n* = 7 (5.1%)
Bogic M et al., 2012 (28)	*n* = 854 [*n* = 416 (48.7%) males, *n* = 438 (51.3%) females, 18–65]	former Yugoslavia	Germany, Italy, UK	Cross-sectional, random/non-random sampling	Pre-war factors, war factors, post-war factors	LSC-R	Anxiety, depression, PTSD	MINI	Age, gender, marital status, education, employment status, number of pre-war, war traumatic events, time since the most traumatic event, host language fluency, residence status, country of residence	Own scales	Anxiety: *n* = 74* (8.7%, SE = 1.0), depression: *n* = 293* (34.3%, SE = 1.6), PTSD: *n* = 283* (33.1%, SE = 1.6)
Carta MG et al., 2018 (29)	*n* = 409 [*n* = 179 males (44%), *n* = 230 females (56%), ≥18]	Mali	Burkina Faso	Cross-sectional, random sampling	War	–	Anxiety and mood disorders, PTSD	SSS-PTSD, K6 scale	Age, gender, death of a family member, severe problems with food, injury or physical damage to self or acquaintances, difficulties related to housing	Own scale	Anxiety and mood disorders: *n* = 306 (75.0%), males: *n* = 142 (79.8%), females: *n* = 164 (71.3%), PTSD: *n* = 350 (85.6%), males: *n* = 152 (85.4%), females: *n* = 198 (86.1%)
Cengiz I et al., 2019 (37)	*n* = 310 [*n* = 164 males (52.9%), *n* = 146 females (47.1%), ≥18]	Syria	Turkey	Cross-sectional, convenience sampling	War	HTQ	PTSD	IES-R	Age, gender, marital status, education, employment status, having children, family size, monthly income, length of stay in host country, smoking history, alcohol history, wishing to return to home country	Own scale	PTSD: *n* = 248* (80%), M = 18.80 (±7.66), males *n* = 130 (79.3%), females *n* = 118 (80.8%)
Chernet A et al., 2021 (73)	*n* = 107 [*n* = 95 males (89%), *n* = 13 females (11%), ≥16]	Eritrea	Switzerland	Cohort, convenience sampling	Traumatic events	Baseline screening for mental health, resilience	Anxiety, depression, PTSD	PHQ-SADS, PTSD-CL-S	NA	NA	Anxiety: *n* = 11*(10.3%), depression: *n* = 16*(15.0%), PTSD: *n* = 52*(48.6%)
Chung MC & Shakra M, 2022 (38)	*n* = 475 [*n* = 265 males (56%), *n* = 210 females (44%), 18–82]	Syria	Sweden	Cross-sectional, convenience sampling, snowball sampling	Traumatic events	HTQ	PTSD	HTQ	Age, gender, marital status, education, time since leaving home country, duration of stay in host country	Own scale	PTSD: *n* = 123 (26%), M = 50.13 (SD = 8.10), males: M = 34.81 (SD = 12.41), females: M = 34.42 (SD = 2.15)
Cheung MC et al., 2018 (39)	*n* = 1,197 [*n* = 715 males (60%), *n* = 482 females (40%), ≥18]	Syria	Sweden, Turkey	Cross-sectional, convenience sampling	Traumatic events	HTQ	PTSD	HTQ	Education, country lived in, residence location	Own scale	PTSD: *n* = 515* (43%)
Chung MC et al., 2018 (40)	*n* = 564 [*n* = 381 males (67.55%), *n* = 183 females (32.44%), ≥18]	Syria	Sweden	Cross-sectional, convenience sampling	War traumatic events	CES, HTQ	PTSD	HTQ	Age, gender, marital status education	Own scale	PTSD: *n* = 169* (30%), M = 15.59 (SD = 5.56)
Dietrich H et al.,2019 (41)	*n* = 175 [*n* = 153 males (87%), *n* = 12 females (13%), 18–24.9]	Syria, Iraq	Germany	Cross-sectional, cluster-based total population sampling	Civil war	Own scale	PTSD	ETI, SSS-PSD, SCL-10	Gender, education, social origin, living conditions	Own scale	PTSD: *n* = 14 (8%), 95% CI: 3.9–12.1 (with ETI: 9.5%, with SSS–PSD: 6.1%)
Eiset AH et al., 2022 (42)	*n* = 712: *n* = 113 Denmark, *n* = 599 Lebanon [*n* = 222 males (27%), *n* = 490 females (73%), ≥18]	Syria	Lebanon, Denmark	Cross-sectional, one-stage cluster random sampling	Long distance migration	Adapted from Spolaore and Warcziarg distance estimates	PTSD	HTQ	Age, gender, exposure to violence during migration, socioeconomic status, general mental well-being	WHO-5	PTSD: Denmark *n* = 68* (60.2%), Lebanon *n* = 330*(55.1%)
Ersahin Z, 2020 (43)	*n* = 805 [*n* = 329, males (41%), *n* = 383 females (59%), 19–77]	Syria	Turkey	Cross-sectional, convenience sampling	Civil war	HTQ-14	PTSD	IES-R	Age, gender, marital status, duration of stay in host country	Own scale	PTSD: *n* = 668*(83%), M = 41.25 (SD = 18.04)
Familiar I et al., 2021 (44)	*n* = 580 Women, (≥18)	Democratic Republic of Congo	Uganda	Cross-sectional, respondent-driven sampling	Sexual, non-sexual violence	HTQ	Depression, PTSD	PHQ-2, HTQ-Part 1	Age, marital status education, level of social support	Own scale	Depression: *n* = 330* (57%), 95% CI: 51–63, PTSD: *n* = 423*(73%), 95% CI: 67–78
Feyera F et al., 2015 (45)	*n* = 847 [*n* = 383 males (53.9%), *n* = 448 females (53.9%), ≥18]	Somalia	Ethiopia	Cross-sectional, multistage probability sampling	Trauma events, basic needs	HTQ	Depression	PHQ-9	Gender, marital status, housing status, witnessing the murder of a family/friend, cumulative traumatic events	Own scale	Depression: *n* = 324* (38.3%), 95% CI: 34.9–41.9, males: *n* = 101 (11.92%), females: *n* = 187 (22.08′%)
Garoff F et al., 2021 (21)	*n* = 784 [*n* = 473 males (60%), *n* = 311 females (40%), ≥18]	>3 countries^2^	Finland	Cross-sectional, population-based sampling	PTEs	10 items adapted from HTQ	Anxiety, depression	HSCL-25	Age, gender	Own scale	Anxiety: *n* = 268* (34.2%), 95% CI: 32.1–36.2, Depression: *n* = 327* (41.7%), 95% CI: 39.6–43.9
Gottvall M et al., 2019^**^ (46)	*n* = 1,215 [*n* = 763, males (62.8%), *n* = 452 females (37.2%), 18–64]	Syria	Sweden	Cross-sectional, random sampling	Torture	RTHC	PTSD	HTQ	Gender, social support	Own scale	PTSD: *n* = 372* (30.6%)
Jeon BH et al., 2009 (47)	*n* = 367 [*n* = 151 males (41%), *n* = 216 females (59%), >20]	North Korea	South Korea	Cross-sectional, convenience sampling	Trauma events	GHQ	Depression	CES-D	Age, gender, marital status, education, employment status, residence, religion, subjective health status, health behavior	Own scales	Depression: *n* = 88 (24%), males: *n* = 36 (23.8%), females: *n* = 52 (24.1%)
Kaya E et al., 2019 (48)	*n* = 420 [*n* = 183, males (43%), *n* = 237 females (57%), ≥18]	Syria	Turkey	Cross-sectional, convenience sampling	War	HTQ	Depression, PTSD	BDI, HTQ	Age, marital status, education, duration of asylum in Turkey, past psychiatric disorder	Own scale	Depression: *n* = 200 (47.7%) (HTQ: M = 2.18 (SD = 0.55), BDI: M = 20.49 (SD = 10.62)), PTSD: *n* = 153 (36.5%)
Kazour F et al., 2017 (49)	*n* = 452 [*n* = 200 males (44.2%), *n* = 252 females (55.8%), 18–65]	Syria	Lebanon	Cross-sectional, convenience sampling	Exposure to trauma	MINI (Arabic version)	PTSD	MINI (Arabic version)	Age, gender, marital status, education, employment status, duration of displacement	Own scales	PTSD: *n* = 123 (27.2%), 95% CI: 23.1–31.3, males: *n* = 55 (44.7%), females: *n* = 68 (55.3%)
Kim HH et al., 2011 (50)	*n* = 144 [*n* = 20 males (14%), *n* = 124 females (86%), 21–75]	North Korea	South Korea	Cross-sectional, convenience sampling	–	–	Anxiety, depression	SCL-90-R, CES-D	Age, gender, marital status, employment status, monthly income, history of physical illness, escape duration	Own scales	Anxiety: *n* = 49 (34.0%), males: *n* = 2 (10%), females: *n* = 47 (37.9%), depression *n* = 56 (38.9%), males: *n* = 5 (25%), females: *n* = 51 (41.1%)
Kim I, 2018 (51)	*n* = 184 [*n* = 77, males (42%), *n* = 107 females (58%), 18–87]	Burma	USA	Cross-sectional, convenience sampling	Torture, trauma, forced displacement	–	Anxiety, depression, PTSD	RHS-15, HSCL-10, 15 item	Age, gender, marital status, education, ethnicity, duration of stay in camp, duration of stay in host country, English proficiency	Own scale	Anxiety: *n* = 37* (20.3%), depression: *n* = 39* (21.2%), PTSD: *n* = 93* (50.4%)
Kira IA et al., 2017 (52)	*n* = 196 [*n* = 134 males (68.4%), *n* = 62 females (31.6%), 18–63]	Syria	Egypt	Cross-sectional, snowball sampling	War trauma, stress	CTS-S	PTSD	CAPS-2	NA	NA	PTSD: *n* = 66* (33.7%), M = 24.18 (SD = 19.25)
Lamkaddem M et al., 2014 (72)	*n* = 410 [*n* = 241 males (58.8%), *n* = 169 females (41.2%), ≥18]	Afghanistan, Iran, Somalia	Netherlands	Longitudinal, random sampling	Traumatic events	HTQ-part 1	PTSD	HTQ-part 4	Age, gender, mental health care utilization, PTSD score	NA	PTSD: *n* = 28 (16.3%), M = 1.81 (SD = 0.68)
Lee YJ et al., 2016 (53)	*n* = 177, [*n* = 48 males (27.12%), *n* = 129 females (72.88%)]	North Korea	South Korea	Cross-sectional, convenience sampling	Traumatic events in North Korea	Trauma Exposure Check List for North Korean FDPs	Depression, PTSD	CES-D, IES-R	Age, gender	Own scale	Depression: *n* = 82 (46.33%), PTSD: *n* = 71 (40.11%)
Lenferink L I.M et al., 2022 (27)	*n* = 613 [*n* = 326, males (53%), *n* = 287 females (47%), ≥18]	>3 countries^3^	Australia	Longitudinal, snowball sampling	PTE, PMLD	HTQ	Depression, PTSD	PDS-17, PHQ-9, PMLD-CL	Gender, age, trauma count, difficulties relating to housing, not enough money to buy food, pay the rent and bills, or buy necessary clothes, not being able to find work, separation from family, worry about family back home, difficulties accessing treatment for health or mental health problems	NA	Depression: *n* = 156 (25.4%), PTSD: *n* = 42 (7.2%)
Lin SL et al., 2020 (22)	*n* = 29,670 [*n* = 272 refugees: 145 males (53.3%), *n* = 127 females (46.7.%), 45–85]	>3 countries^4^	Canada	Cross-sectional, random sampling	Refugee status	Self-reported information	Depression	CESD 10	Age, gender, marital status, education, employment status, household income, health status, social connections	Own scale	Depression: *n* = 60* (22.1%)
Maharaj V et al., 2017 (23)	*n* = 335 [*n* = 178 males (53.1%), *n* = 157 females (46.9%), 18–75]	>3 countries^5^	South Africa	Cross-sectional, convenience sampling	Food insecurity	12-Month Food Security Scale-SF	Anxiety, depression	HSCL-25	Age, gender, marital status, education, employment status, monthly income, migration status, social support, racism	Own scale	Anxiety: *n* = 165 (49.4%), depression *n* = 180 (54.6%)
Mahmood HN et al., 2019 (54)	*n* = 988 [*n* = 494 males (50%), *n* = 494 females (50%)]	Syria	Iraq	Cross-sectional, stratified random sampling	War	WAEC-created based on the existing trauma instruments-WES, LEC-5 for DSM-5	Depression, PTSD	DSM-5 PCL-5, Kurdish Kurmanji, Arabic version of D-HSCL-15	Age, gender, marital status, education, employment status, duration of stay in camp, area in which participants were grown up	Own scale	Depression: *n* = 586* (59.4%), M = 29.36 (SD = 8.52), PTSD: *n* = 606* (61.4%), M = 26.44 (SD = 15.3)
Mwanamwambwa V & Pillay BS, 2021 (55)	*n* = 267 [*n* = 128 males (47.94%), *n* = 139 females (52.06%), 18–65]	Rwanda	Zambia	Cross-sectional, purposive sampling, snowball sampling	Genocide	IES-R	PTSD	IES-R, GHQ-28	Gender, marital status, number of children, education, employment status, financial support	Own scale	Depression: *n* = 60* (22.8%), PTSD: *n* = 205* (76.8%)
Naal H et al., 2021 (56)	*n* = 3,255 [*n* = 1,071 males (33%), *n* = 2,184 females (67%), ≥18]	Syria	Lebanon	Cross-sectional, random sampling	Traumatic events	Baseline screening	Depression	PHQ-2, 9	Age, gender, marital status	Own scale	Depression: *n* = 1510* (46.4%)
Naja W et al., 2016 (57)	*n* = 310 [*n* = 120 males (38.7%), *n* = 190 females (61.3%), ≥18]	Syria	Lebanon	Cross-sectional, random sampling	Religiosity	Original Arabic religiosity scale	Depression	MINI	Age, gender, religiosity	Own scale	Depression: *n* = 136 (43.9%), 95% CI: 38.5–49.4
Nam B et al., 2016 (58)	*n* = 304 [*n* = 102 males (33.8%), *n* = 200 females (66.2%), ≥18]	North Korea	South Korea	Cross-sectional, snowball sampling	Family cohesion	FACES-III, Korean version	Depression	CES-D, Korean version	Age, gender, resilience, time spent in South Korea	Own scale, K-CD-RISC	Depression: *n* = 135 (44.4%)
Nesterko Y et al., 2019 (24)	*n* = 502 [*n* = 348 males (69%), *n* = 154 females (31%), ≥18]	>3 countries^6^	Germany	Cross-sectional, cluster-based total population sampling	Traumatic events, flight related experiences	DSM-5 LEC-5	Anxiety, depression, PTSD	PCL-5, PHQ-9, HSCL-25	NA	NA	Anxiety: 210* (41.8%), depression: *n* = 108* (21.6%), PTSD: *n* = 174* (34.7%)
Nickerson A. et al., 2009 (59)	*n* = 315 [*n* = 150 males (47.5%), *n* = 165 females (52.5%)]	Iraq	Australia	Cross-sectional, convenience sampling	Past traumatic experiences, current resettlement difficulties, human rights violations	PMLD, HTQ, HSCL-25, depression subscale	PTSD, depression	HTQ, HSCL-25, depression subscale	Life experiences, fear of extinction	Own scale	Depression: *n* = 107 (34%), PTSD: *n* = 72 (22.9%),
Nissen A et al., 2021 (60)	*n* = 902 [582 males (64.5%), 320 females (35.5%), 18–65]	Syria	Norway	Cross-sectional, random sampling	Potentially traumatic experiences (PTEs), length of flight	RTHC	Anxiety, depression, PTSD	HTQ, HSCL	Age, gender, marital status, education, refugee status, arrival with family/friends, prior family in Norway, length of flight, time in Norway	Own scale	Anxiety: *n* = 271*(30.1%), 95% CI: 25.7–34.9, depression: *n* = 46*(5.2%), 95% CI: 40.6–49.8, PTSD: *n* = 267*(29.7%), 95% CI: 25.4–34.4,
Poole DN et al., 2021 (61)	*n* = 135 [*n* = 80 males (59.26%), *n* = 55 females (40.74%), 18–61]	Syria	Greece	Cross-sectional, purposive sampling followed by systematic sampling	Asylum process	–	Depression	PHQ-8	Gender, marital status, number of children, length of asylum process	NA	Depression: *n* = 59* (44%)
Rasmussen A et al., 2012 (25)	*n* = 660 [*n* = 345 males (52.3%), *n* = 315 females (47.7%), 18–97]	>3 countries^7^	USA	Cross-sectional, multistage area probability sampling	Traumatic events, migration	NLAAS measures	Depression, PTSD	WMH-CIDI	Gender, country of origin, time in host country, use of mental health services	Own scale	Depression: *n* = 97 (14.74%, SE = 0.20), PTSD: *n* = 31 (4.75%, SE = 0.16)
Sagaltici E et al., 2019 (62)	*n* = 342 [*n* = 163 males (47.66%), *n* = 179 females (52.34%), 18–65]	Syria	Turkey	Cross-sectional, random sampling	War	Own scale (based on stressful life events screening questionnaire)	PTSD	DSM-IV-TR	Age, gender, number of traumatic events	Own scale	PTSD: *n* = 106* (31%)
Silove D et al., 2010 (63)	*n* = 126 [*n* = 49 males (31%), *n* = 77 females (61%), 18–88]	Bosnia	Australia	Cross-sectional, convenience sampling	War	Own scale	Depression, PTSD	CAPS, SCID, ASA-SI	Age (at the time of entering Australia), gender	Own scale	Depression: *n* = 58 (46%), PTSD: *n* = 79 (63%)
Taylor EM et al., 2014 (64)	*n* = 366 [*n* = 218 males (60%), *n* = 144 females (40%), 18–84]	Iraq	USA	Cross-sectional, random sampling	War trauma	–	Anxiety, depression, PTSD	HSCL 25, PC-PTSD	Age, marital status, employment status, duration of stay in host country, health behavior	Own scale	Anxiety: *n* = 182 (50%), depression: *n* = 177 (49%), PTSD: *n* = 112 (31%)
Tekeli-Yesil S et al., 2018 (65)	*n* = 285 [*n* = 144 males (51%), *n* = 141 females (49%), ≥18]	Syria	Turkey	Cross-sectional, convenience, snowball sampling	War experience	Own questionnaire adapted from HTQ, PMLD-CL	Anxiety, depression, PTSD	MINI (Arabic version)	Age, gender, marital status, employment status, economic status, family unity, size of the family during pre-and post-migration	Own scale	Anxiety: 151* (53.2%), depression: *n* = 151* (52.8%), PTSD: *n* = 152* (53.2%)
Tekin A et al., 2016 (66)	*n* = 238 [*n* = 105 males (44.1%), *n* = 133 females (55.9%), 18–65]	Iraq	Turkey	Cross-sectional, random sampling	Lifetime exposure to traumatic events	Stressful life events screening questionnaire	Depression, PTSD	SCID-I	Age, gender, marital status, education, employment status, duration of displacement, psychiatric or other medical history	Own scale	Depression: *n* = 94 (39.5%), PTSD: *n* = 102 (42.9%)
Tinghög P et al., 2017^**^ (67)	*n* = 1,215 [*n* = 763 males (63%), *n* = 452 females (37%), 18–64]	Syria	Sweden	Cross-sectional, random sampling	Refugee-related PTEs	Own scale	Anxiety, depression, PTSD	HSCL-25, HTQ	Age, gender, marital status, education, year of arrival in host country	Own scale	Anxiety: *n* = 386*(31.8%), 95% CI: 29.2–34.7, depression: *n* = 488* (40.2%), 95% CI: 36.9–43.3, PTSD: *n* = 363* (29.9%), 95% CI: 27.2–32.6
Vonnahme LA et al., 2015 (68)	*n* = 386 [*n* = 204 males (52.85%) *n* = 182 females (47.15%), 18–83]	Bhutan	USA	Cross-sectional, random sampling	Traumatic events	HTQ	Anxiety, depression, PTSD	HSCL-25, HTQ	Age, gender, residence status	Own scale	Anxiety: *n* = 69 (18%), depression: *n* = 80 (21%), PTSD: *n* = 14 (4%)
Winkler JG et al., 2018 (26)	*n* = 650 [*n* = 486 males (74.8%), *n* = 164 females (25.2%), ≥18]	>3 countries^8^	Germany	Cross-sectional, cluster-based total population-based sampling	Asylum procedure, legal situation	Own scale	Anxiety, depression, PTSD	HSCL-25, PDS	Age, gender, country of origin, duration of stay in host country	Own scale	Anxiety: 340* (52.3%), depression: *n* = 399* (61.3%), PTSD: 271* (41.7%)
Yang MS & Mutchler JE, 2020 (69)	*n* = 127 [*n* = 30 males (24%), *n* = 97 females (76%), 55+]	China	USA	Cross-sectional, convenience sampling	Hmong refugee status	Own scale	Depression	HSCL-10	Age, gender, marital status, education, employment status, English proficiency	Own scale	Depression: *n* = 91.44* (72%), M = 2.23 (SD = 0.67)
Yun S et al., 2021 (70)	*n* = 219 [*n* = 219 females (100%), 23–58]	Iraq	USA	Cross-sectional, random sampling	Acculturative stress, traumatic events	SAFE, HTQ	Anxiety, depression	HSCL-25	Age (when left Iraq), marital status, education, financial capacity, spoken language, prior mental illness	Own scale	Anxiety: 100* (45.6%), M = 1.67 (SD = 0.76), depression: *n* = 121* (55.3%), M = 1.69 (SD = 0.62)

^A,B^Studies conducted using the same sample; *Prevalences calculated manually; ^**^Studies conducted using the sample with different outcomes; CI, Confidence Interval; NA, Not Available; M, Mean; SD: Standard deviation; SE: Standard error; ASA-SI, The Adult Separation Anxiety-Structured Interview; BDI, Beck Depression Inventory; BDI-II, Becks depression inventory–II; CAPS, Clinical Administered PTSD Scale; DASS-21, CES, Centrality of event scale; CES-D, Center for Epidemiologic Studies Depression Scale; CESD 10, Center for Epidemiologic Studies Depression 10; Depression anxiety stress scale; DSM-5, Diagnostic and Statistical Manual of Mental Disorders Fifth Edition; DSM-IV-TR, The Diagnostic and Statistical Manual of Mental Disorders, fourth edition, text revision; ETI, Essen Trauma Inventory; FACES III, Family Adaptability and Cohesion Scale III; GAD-7, General Anxiety Disorder-7; GHQ, General Health questionnaire; HSCL-25, Hopkins Symptom Checklist; HTQ, The Harvard Trauma Questionnaire; HTQ-16, Harvard Trauma Questionnaire-16; IES-R, Impact Event Scale-Revised; K6 scale, Kessler K6 scale; LEC-5, Life Events Checklist-5; LSC-R, Life Stressor Checklist-Revised; MDD, Major Depressive Disorder; MEV, My exposure to violence; MSPSS, Multidimensional Scale of Perceived Social Support; MINI, Mini International Neuropsychiatric Interview; NLAAS, The National Latino and Asian American Survey; OSSS-3, Oslo-3 Social Support Scale; PHQ-SADS, Patient Health Questionnaire Somatic Anxiety and Depression Syndrome; P-CTD, The Post-Cumulative Trauma-Related Disorders Measure; PC-PTSD, Primary Care PTSD Screen; PDS, Posttraumatic stress diagnostic scale; PHQ-8, Patient Health Questionnaire-8; PTEs, Potentially Traumatic Experiences; PMLD, Post migration living difficulties; PMLD-CL, Postmigration Living Difficulty Checklist; RTHC, Refugee Trauma History Checklist; SAFE, The Social, Attitudinal, Familial and Environmental Acculturative Stress; SCID-I, Structured Clinical Interview for DSM-IV; SCL-90-R, Symptom checklist-90-R; SPT-NK, Scale for Past Traumas in North Korea; SPT-D, Scale for Past Traumas during Defection; SSS-PSD, Short Screening Scale for Posttraumatic Stress Disorder; PTSD-SS, PTSD-Symptom Scale; WAEC, War and Adversity Exposure Checklist; WES, War Exposure Scale; WTSS, War trauma screening scale; WHODAS 2.0: World Health Organization Disability Assessment Schedule 2.0; WMH-CIDI, World Mental Health Composite International Diagnostic Interview; GAD, Generalized anxiety disorder; PTSD, Posttraumatic stress disorder; MDD, Major depressive disorder; USA, United States of America.

1 Congo, Burundian, Somali, Rwanda, Ethiopia, Eritrea, South-Sudan, Sudan, Kenya, Senegal.

2 Russia and former Soviet union, Middle East and North Africa (Turkey, Iran, Iraq), Africa excluding North Arica (Somalia, Nigeria, Angola, Cameroon), Others (Nicaragua, Albania, Bangladesh, India, Cuba, Kosovo, Sri Lanka).

3 Iraq, Syria, Iran, Afghanistan, Sri Lanka, Burma, Pakistan, Other.

4 China, Haiti, Hungary, Jews from Central Europe, Chile, Lebanon, Baltic origins, Ukraine, Vietnam, South Asians from Uganda, Iran, Afghanistan, Bangladesh, Yugoslavia, Palestinian Arabs from Palestine or Israel, Cambodia, Iraq.

5 Democratic Republic of Congo, Zimbabwe, Burundi, Ghana, Malawi, Mozambique, Rwanda, Uganda.

6 Cameroon, Eritrea, Iraq, Nigeria, Syria, Turkey, Venezuela, and refugees from 40 countries.

7 Puerto Rica, Mexico, Cuba, all other Latins, Philippines, China, Vietnam, Asian.

8 Syria, Afghanistan, Iraq, Albania, Iran, Moldova, Serbia, Kosovo, Russian Federation, Pakistan, Eritrea, others.

Country of origin of FDPs varied: with FDPs from Africa [Congo (31, 44), Eritrea (35, 73), Mali (29), Rwanda (55), Somalia (45, 72)]; eight studies from Asia [Bhutan (68), Cambodia (36), China (69), North Korea (47, 50, 53, 58), Sri Lanka (34)]; 28 studies from Middle East [Iran (72), Iraq (41, 54, 59, 64, 66, 70), Syria (30, 32, 33, 37–43, 46, 48, 49, 52, 54, 56, 57, 60–62, 65, 67, 71, 74), Afghanistan (72)], and two studies from Europe (Bosnia (63), former Yugoslavia (28)). The age range of participants ranged from 18 years to 97 years. All studies included male and female participants (49.83% vs. 50.17%) except one study which was conducted among Yazidi women in Iraq.

Anxiety, depression and PTSD were reported in 18, 38 and 41 studies, respectively. Most studies used diagnostic measures HTQ (27, 33, 36, 38–40, 42, 44, 46, 48, 60, 67, 68, 72) and HSCL (21, 23, 24, 26, 30, 36, 51, 54, 59, 64, 67–70). Studies also collected data on potential covariates such as socio-demographics (e.g., age, gender, education, marital status, employment status) (21–23, 25–51, 53–58, 60–72), religiosity (57), duration of displacement (49, 66), duration of stay in camp (51, 54), asylum duration (32, 48), family history of mental health diseases (32, 35), personal history of mental health treatment (30), mental health care use (25), difficulty in access to mental health care (27), migration status (23), social support (23, 44, 46), racism and food insecurity (23).

The studies Acarturk et al., 2020 (30) and Fuhr DC et al., 2019 (74) used the same sample and analyzed similar outcomes of anxiety, depression and PTSD. Therefore, only one study was included in the meta-analysis. In the studies by Gottvall et al., 2019 (46) and Tinghög et al., 2017 (67), although study samples were similar, the differences in exposure and outcome measurement scales, study outcomes, and prevalence rates justified the inclusion of both studies in the meta-analysis.

### Anxiety among FDPs in included studies

**Figure 1:**
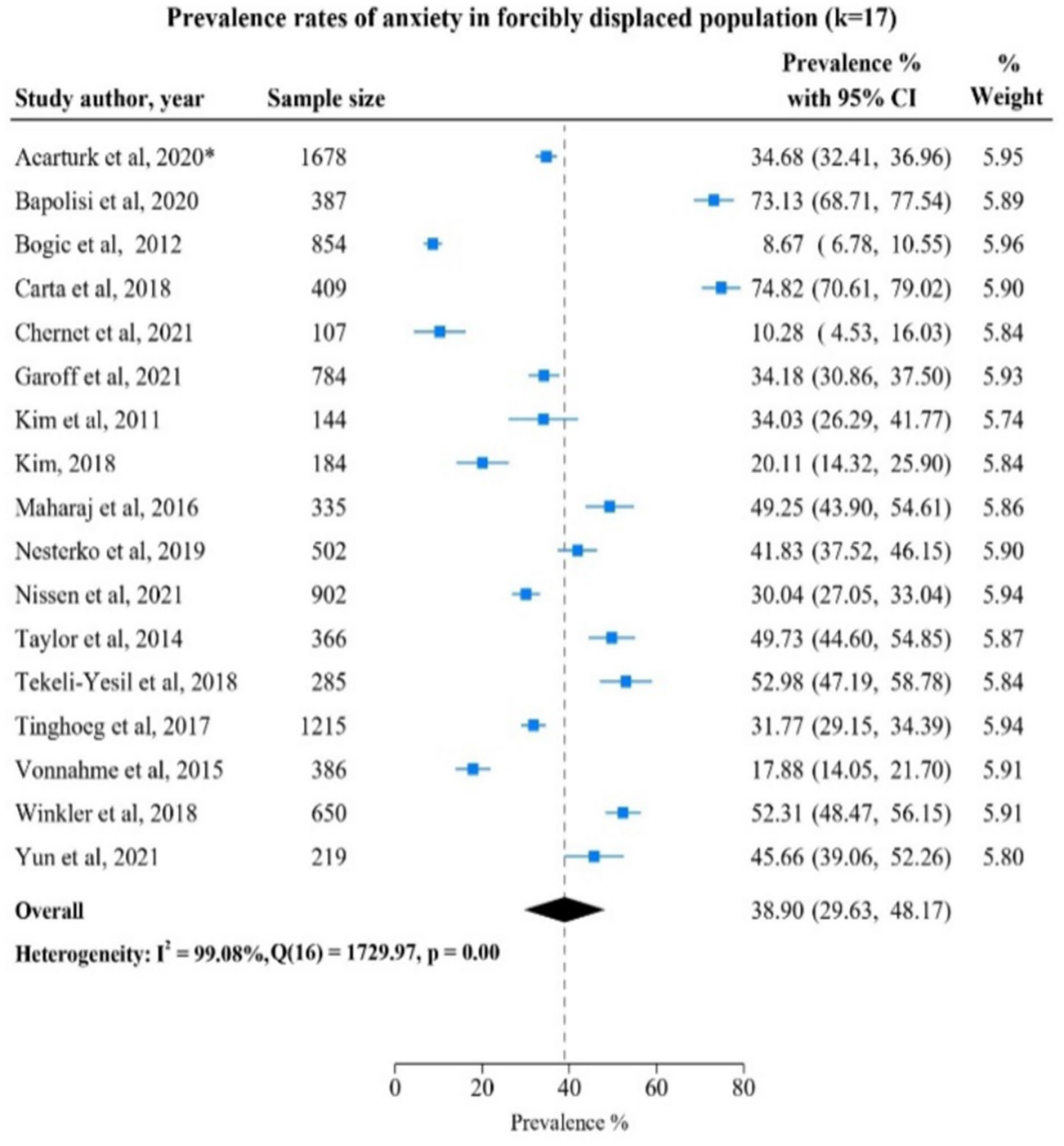
Study authors, year, sample size, anxiety prevalence rate with 95% confidence intervals and random % weight. *Studies (Acarturk et al., 2020, Fuhr et al., 2019) were conducted using the same sample and analyzed the same outcomes, so only one study was considered for meta-analysis.

Studies reporting anxiety disorders were 17 with a total of *n* = 9,407 FDPs. Of these studies, Bogic et al., 2012 (28) found the lowest prevalence rate of 8.67% (95% CI: 6.78; 10.55) in a study of *n* = 854 FDPs from former Yugoslavia living in Germany, Italy, United Kingdom (Figure 1). The highest prevalence of anxiety 74.82% (95% CI: 70.61; 79.02) was reported in a study by Carta et al., 2018 (29) of *n* = 409 FDPs from Mali living in Burkina Faso. The overall prevalence rate was 38.90% (95% CI: 29.63; 48.17) with a substantial heterogeneity of I^2^ = 99.08% between studies included in the meta-analysis.

### Depression among FDPs in included studies

**Figure 2:**
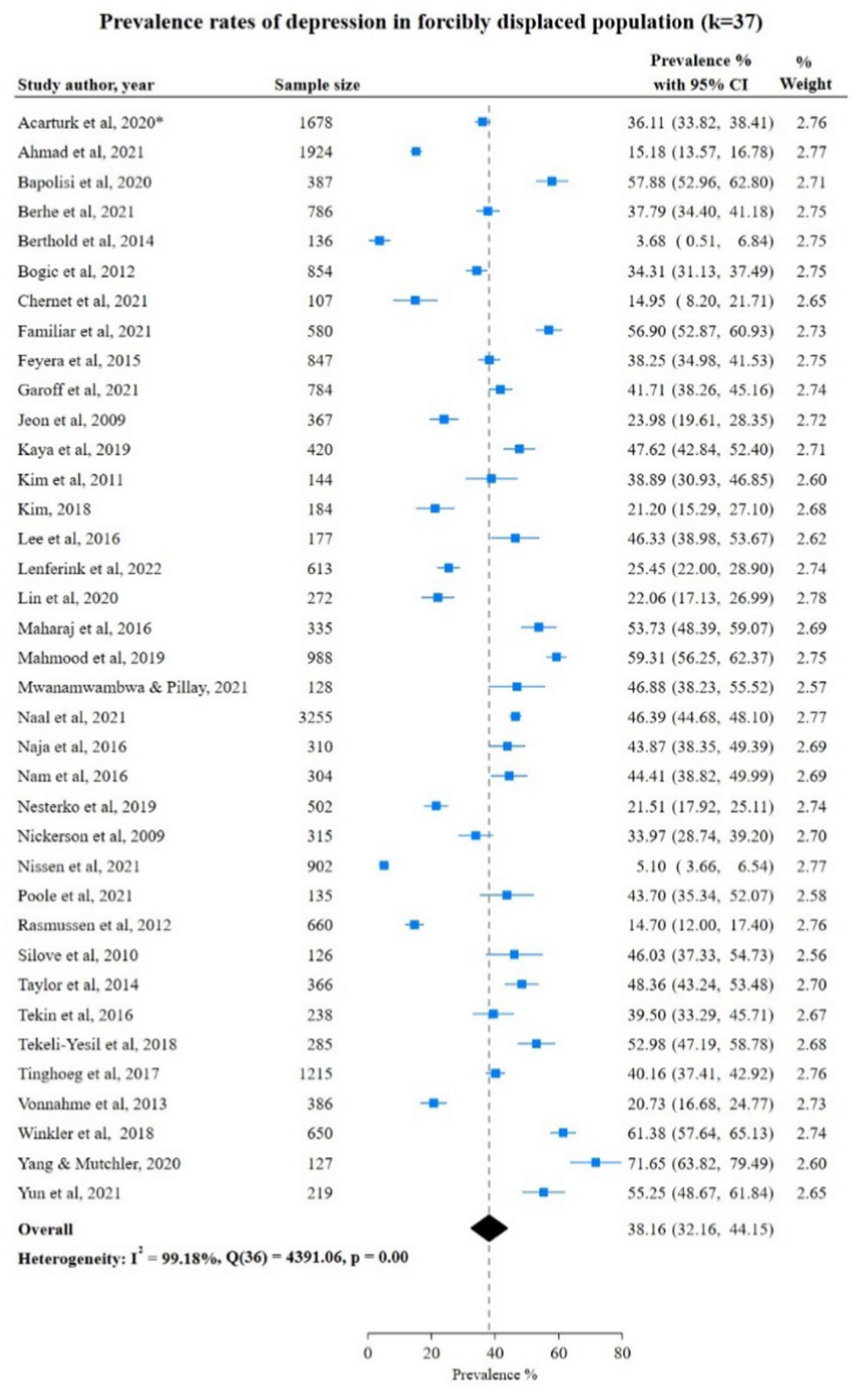
Study authors, year, sample size, depression prevalence rate with 95% confidence intervals and random % weight. ^*^Studies (Acarturk et al., 2020, Fuhr et al., 2019) were conducted using the same sample and analyzed the same outcomes. Therefore, only one study was considered for meta-analysis.

A total of 37 studies investigated depression with a total number of participants of *n* = 21,706. For depression, the range of prevalence rate was broad among included studies reporting the lowest prevalence of 3.68% (95% CI: 0.51; 6.84) in a study Berthold et al., 2014 (36) of *n* = 136 FDPs from Cambodia to the highest prevalence rate of 71.65% (95% CI: 48.67; 79.49) in a study from Yang & Mutchler, 2020 (69) of *n* = 127 FDPs from China. The overall pooled prevalence rate was 38.16% (95% CI: 32.16; 44.15) showing a high heterogeneity of I^2^ = 99.18% (Figure 2).

### PTSD among FDPs in included studies

**Figure 3:**
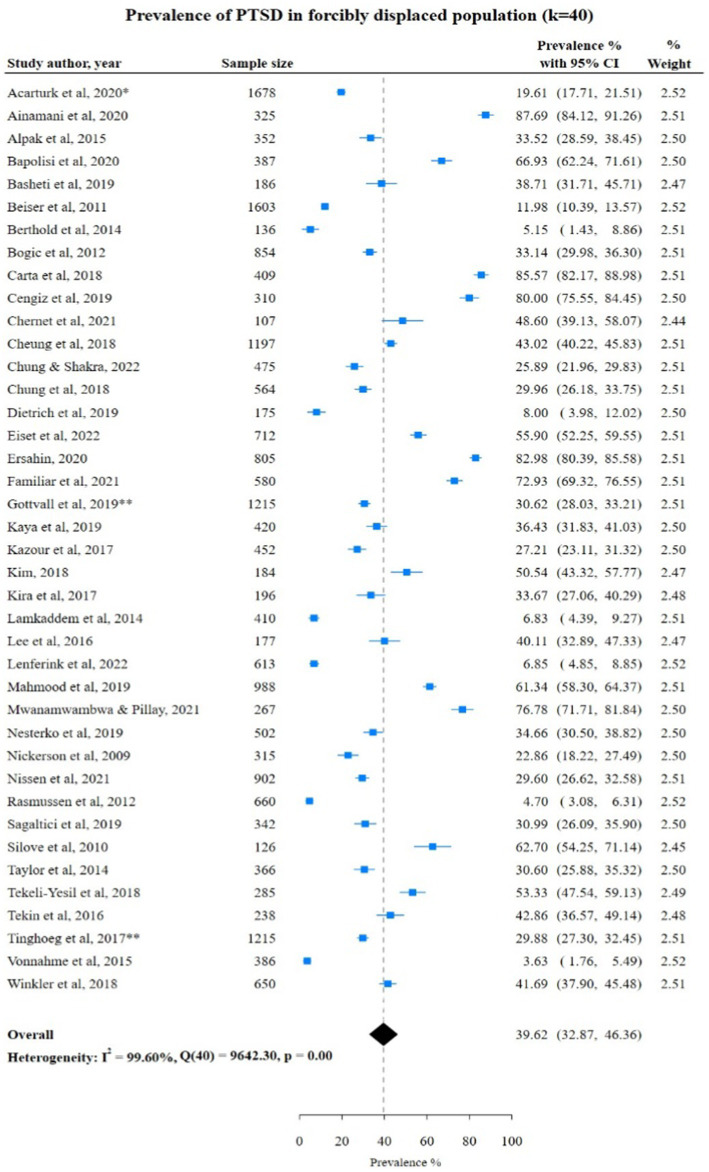
Study authors, year, sample size, PTSD prevalence rate with 95% confidence intervals and random % weight. ^*^Studies (Acarturk et al., 2020, Fuhr et al., 2019) conducted using the same sample and analyzed the same outcomes. Therefore, only one study was considered for meta-analysis; **Studies conducted using the same sample with different outcomes.

Overall, 40 studies reported PTSD in a study population *n* = 21,764 of which 8,007 forcibly displaced populations were diagnosed with PTSD with a pooled prevalence rate of 39.62% (95% CI: 32.87; 46.36). The lowest PTSD prevalence rates were reported in studies by both Rasmussen et al., 2012 (25) (*n* = 345) and Berthold et al., 2014 (36) (*n* = 136) with prevalence rates of 4.70% (95% CI: 3.08; 6.31) and 5.15% (95% CI: 1.43; 8.86) respectively. The highest prevalence rate of 87.68% (95% CI: 84.12; 91.26) was found in a study by Ainamani et al., 2020 (31) of *n* = 325 FDPs from DRC. There was substantial heterogeneity between studies reporting PTSD (I^2^ = 99.60%; Figure 3).

### Subgroup analysis by study and sample characteristics

#### Human rights violations by GPI ranking and mental conditions among FDPs

**Figure 4:**
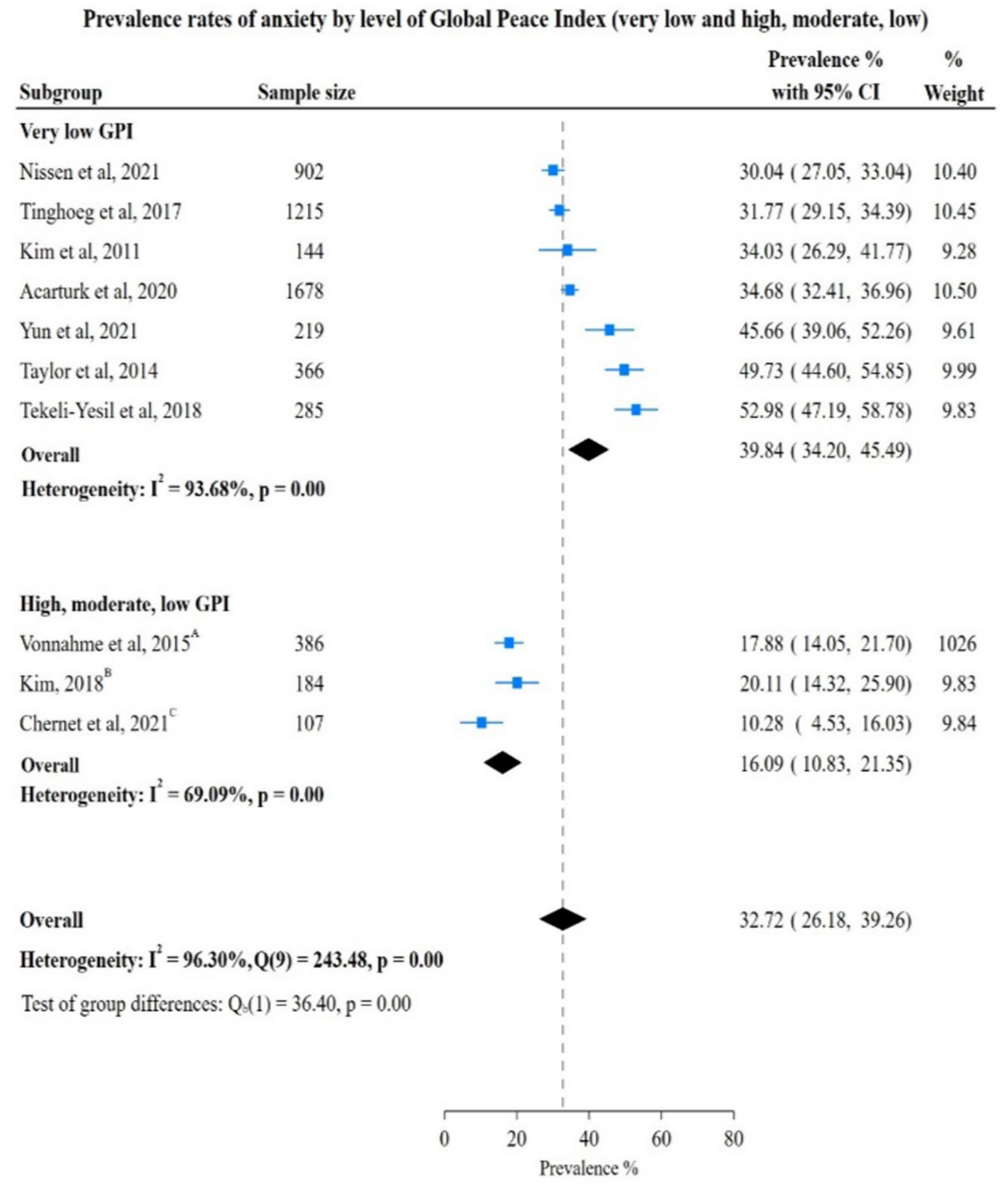
Study authors, year, sample size, anxiety prevalence rates with 95% confidence intervals and random % weight by very low versus all other categoreis of GPI. GPI, Global Peace Index; ^A^High GPI; ^B^Moderate GPI; ^C^Low GPI.

For countries with very low GPI, the prevalence of anxiety was 39.84% (95% CI: 34.20; 45.49) compared to countries with high, moderate and low GPI, where the prevalence of anxiety was 16.09% (95% CI: 10.83; 21.35). The test of group difference yielded a Chi-square statistic of 36.40, (*p* = <0.05) indicating a significant difference in anxiety prevalence rates between these two groups (Figure 4).

**Figure 5:**
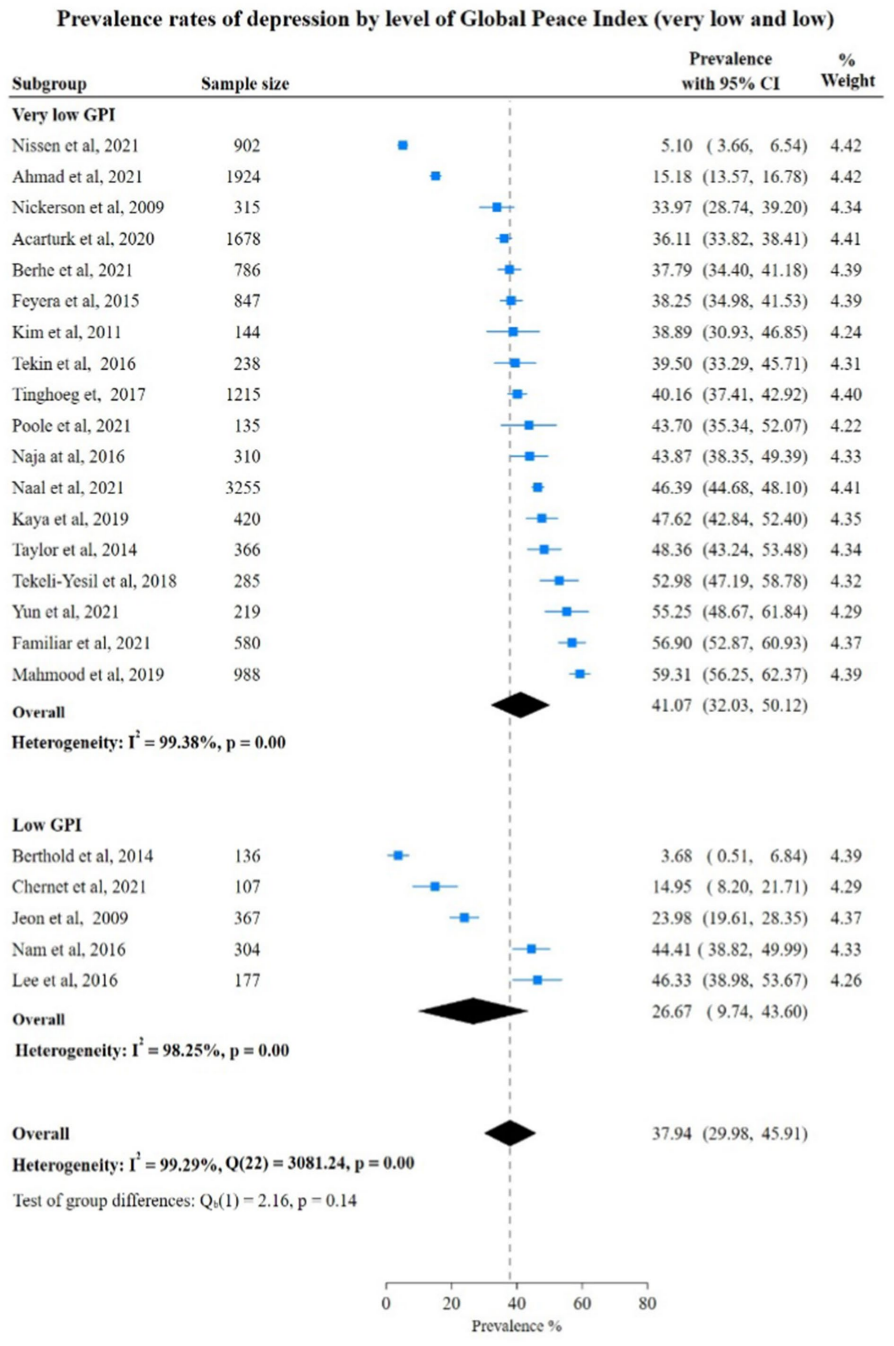
Study authors, year, sample size, depression prevalence rates with 95% confidence intervals and random % weight by very low versus versus low GPI. GPI, Global Peace Index.

**Figure 6:**
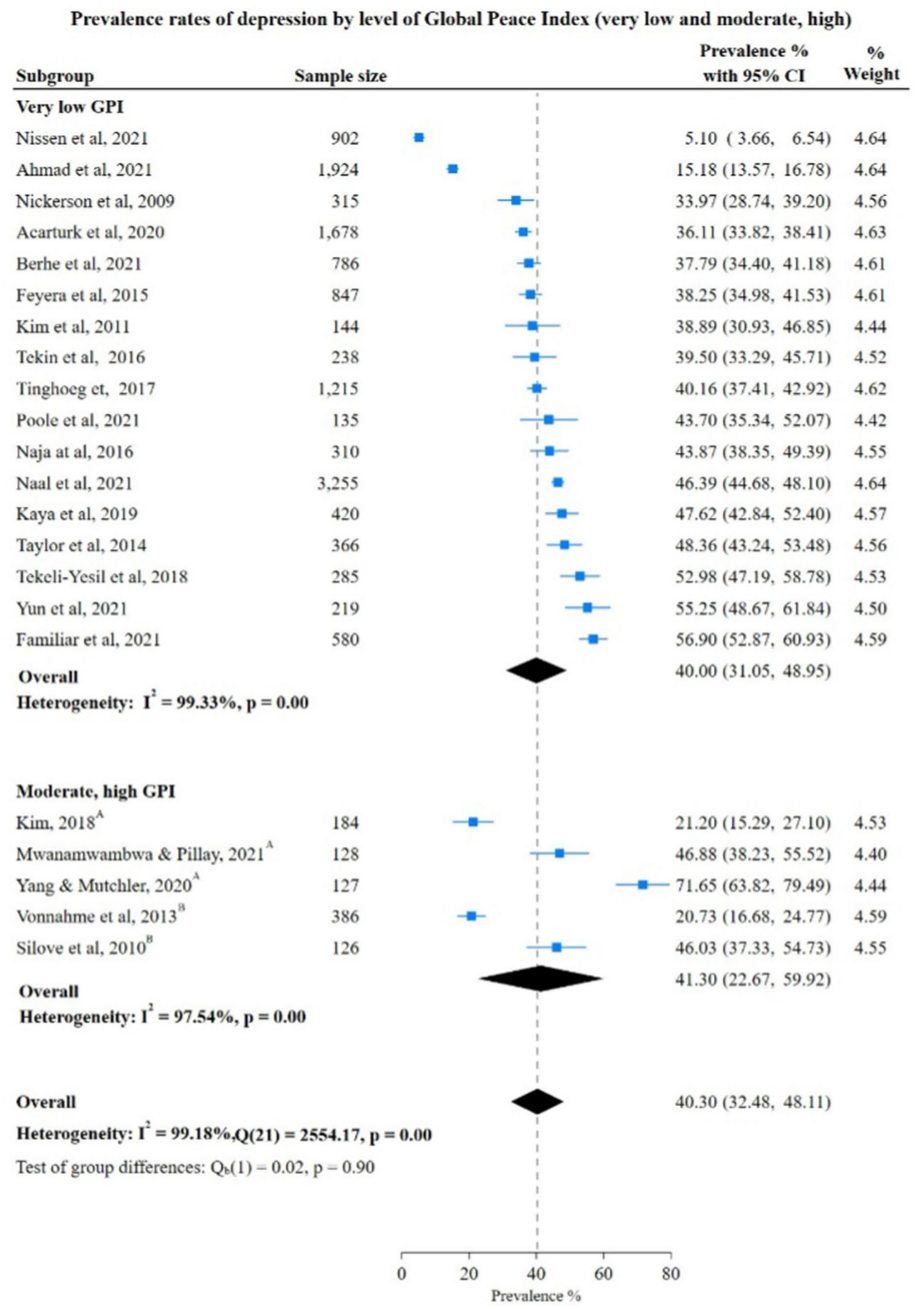
Study authors, year, sample size, depression prevalence rates with 95% confidence intervals and random % weight by very low versus moderate, high categoreis of GPI. GPI, Global Peace Index; ^A^Moderate GPI; ^B^High GPI.

Depression prevalence rate among countries with very low GPI was higher at 41.07% (95% CI: 32.03%; 50.12%) than the pooled rate of depression in low GPI countries at 26.67% (95% CI: 9.74%; 43.60%). The difference in the pooled rate was not significant between very low GPI vs. moderate, high GPI countries 40.00% (95% CI: 31.05; 48.95) vs. 41.30% (95% CI: 22.67; 59.92). However, the test for group differences was not statistically significant [very low vs. low: Chi-square 2.16, (*p* = 0.14); very low vs. moderate and high: Chi-square 0.02, (*p* = 0.90)], showing no significant difference in depression prevalence rates between these subgroups (Figures 5, 6).

**Figure 7:**
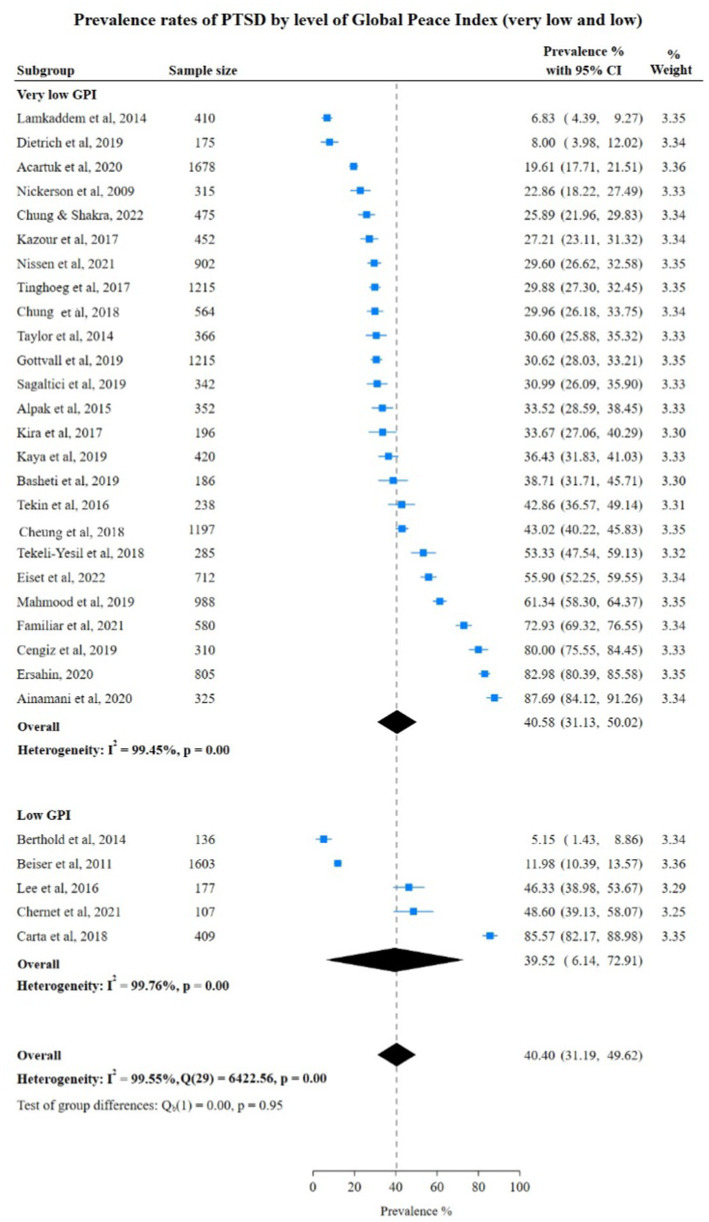
Study authors, year, sample size, PTSD prevalence rates with 95% confidence intervals and random % weight by very low versus low GPI. GPI, Global Peace Index.

**Figure 8:**
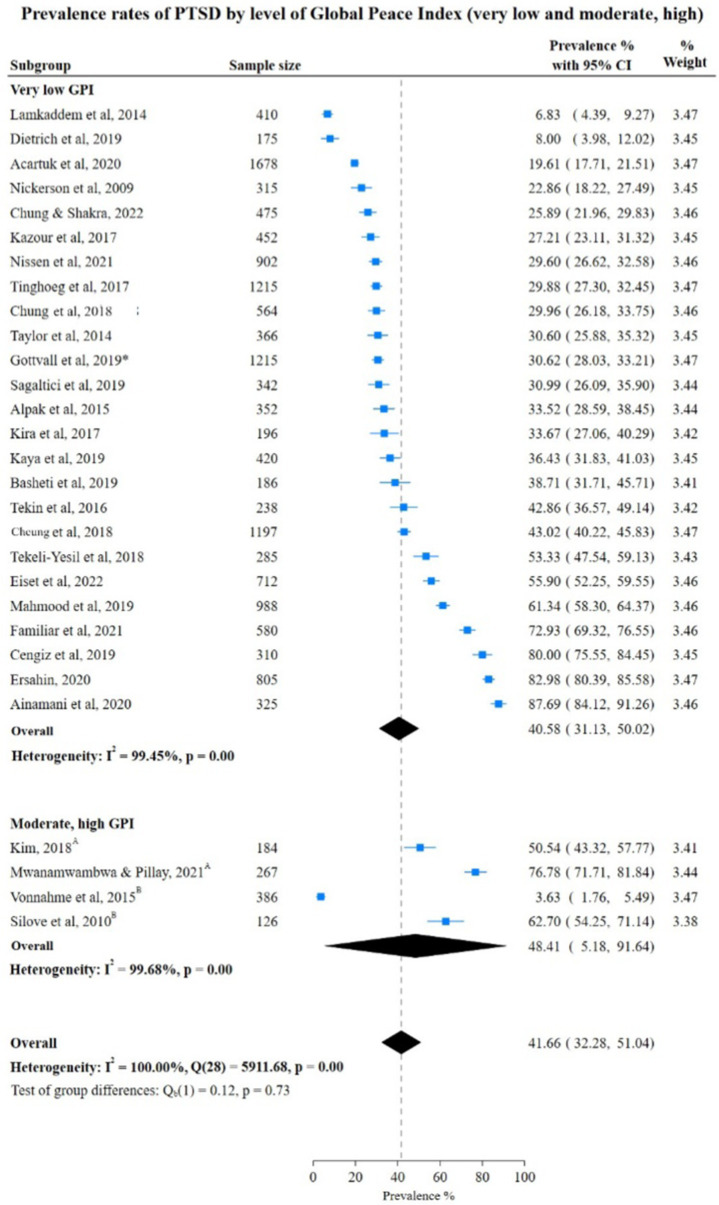
Study authors, year, sample size, PTSD prevalence rates with 95% confidence intervals and random % weight by very low versus low GPI. GPI, Global Peace Index; ^A^Moderate GPI; ^B^High GPI.

In contrast to anxiety and depression, PTSD prevalence rates were higher in countries with moderate and high GPI than in countries with low GPI at 48.41% (95% CI: 5.18; 91.64), 40.58% (95% CI: 31.13; 50.02) respectively. Also, the pooled rate in low GPI countries was 39.32% (95% CI: 6.14%; 72.91%) which did not show a significant difference from the pooled rate of PTSD in very low GPI countries. However, the test for group differences was not statistically significant (very low vs. low: Chi-square 40.40, *p* = 0.95) very low vs. moderate and high: Chi-square 41.66, (*p* = 0.73), indicating no significant difference in PTSD prevalence rates between various subgroups (Figures 7, 8).

### Prevalence rates of mental health conditions in FDPs by risk of bias

We compared prevalence rates of anxiety and depression by risk of bias probability (moderate and low vs. high and very high) (Supplementary Figures 2–6). For anxiety we did not identify differences by risk fo bias subgroups (*p* = 0.74 for anxiety and *p* = 0.30 for depression).

For PTSD, overall prevalence was high in the high risk of bias subgroup at 49.27% (95% CI: 37.18; 61.35). Also, a comparison between the risk of bias subgroups, very high vs. high, revealed a pooled rate of 42.12% (95% CI: 31.66; 52.59) with a Chi-square statistic of 4.55, (*p* = 0.03) indicating a significant difference in PTSD prevalence rates between these two groups. Whereas comparison of the risk of bias subgroups, very high vs. moderate and low, showed a pooled rate of 32.23% (95% CI: 23.39; 41.07) and a non-significant difference in PTSD prevalence rates between these two groups with a Chi-square statistic of 0.42, (*p* = 0.52; Supplementary Figures 7, 8).

### Prevalence rates of mental health conditions in FDPs by study sampling methods

Comparison of prevalence rates of anxiety, depression and PTSD by sampling methods (random and convenience) showed no significant difference: anxiety 40.79% (95% CI: 32.84; 48.75) Chi-square statistic 0.92, (*p* = 0.34); depression 38.27% (95% CI: 32.09; 44.45), Chi-square statistic 0.02 (*p* = 0.88); PTSD 40.01% (95% CI: 31.79; 48.23), Chi-square statistic 0.75, (*p* = 0.39; Supplementary Figures 9–11).

However, observation of prevalence rates by individual sampling method showed that anxiety prevalence rates by random sampling was 44.71% (95% CI: 32.08; 57.35) which was higher than the overall anxiety prevalence of 38.90 (95% CI: 29.63; 48.17). Whereas PTSD prevalence rate was higher by convenience sampling at 42.83% (95% CI: 31.23, 54.44) compared to the overall PTSD prevalence of 39.62% (95% CI: 32.87; 46.36). Similarly, for depression, there was a slight difference in prevalence rate between by the convenience sampling method of 38.67% (95% CI: 30.45; 46.89) and the depression pooled rate of 38.16% (95% CI: 32.16; 44.15).

## Discussion

In this systematic review with meta-analysis, we found pooled prevalence rates of 38.90% for anxiety, 38.16% for depression and 39.62% for PTSD in FDPs. Additionally, the study results also showed a positive association between the level of peace in the country of origin and the mental health conditions of FDPs. For countries with very high human rights violations, prevalence rates of anxiety (39.84%), depression (41.07%) and PTSD (40.58%) were higher compared to countries with low human rights violations. These findings underscore that repeated and enduring human rights violations, rather than isolated traumatic event, contribute substantially to psychopathology and related findings are suggested by studies on the impact of child maltreatment (75, 76).

The experience of multiple traumatic events can be assumed in states of human rights violations. This is in line with studies suggesting that the number of traumatic events is a main predictor of mental health conditions (77, 78). These associations are potentially mediated by neurobiological mechanisms (79) involving changes in the hypothalamic–pituitary–adrenal (HPA) axis activity. Stress events stimulate the HPA axis and sympathetic nervous system (SNS), resulting in an increase in cortisol, alpha-amylase, and heart rate (80), which is positively associated with increased levels of heart rate, mood changes also mental health (81–83).

For the first time ever to the best of our knowledge, we identified an association between the level of human rights violations and the prevalence of anxiety, depression, and PTSD. Human rights violations can be understood as an act of exclusion disrupting the individual’s sense of safety and belonging in society making them more vulnerable to mental health conditions. Human rights violations have been found to be significant determinants of poor mental health in some populations but are outside the usual scope of psychiatric and social epidemiology (84). Few studies have advocated a comprehensive assessment of human rights violation’s impact on health (85, 86). Our findings challenge the traditional understanding of refugee health, which often emphasizes traumatic experiences during forced migration over political and social determinants embodied prior to displacement.

Furthermore, in the subgroup analysis based on risk of bias, studies with moderate and low risk of bias showed higher prevalence rates of anxiety (40.76%). However, the number of studies meeting the criteria for subgroup analysis was limited. On the other hand, for depression and PTSD, studies with a high risk of bias had higher prevalence rates (43.34 and 49.27%) compared to studies with a very high risk of bias. The difference could be due to the higher number of studies qualifying for the high risk of bias category. In the subgroup analysis based on study sampling methods, anxiety prevalence rate was high (44.71%) by random sampling while the prevalence rate of PTSD (42.83%) was high by convenience sampling.

The study is not without limitations, including reliance on self-report measures at a single time point, lack of data on the duration and timing of human rights violations, and potential exclusion of relevant studies.

We did an additional search on individuals fleeing from territories occupied by Russia. However, due to the difficulties in the territories occupied by Russia no study provided data on refugees from these areas. In case of countries occupied it is almost impossible to conduct a representative study. Recent studies on the impact of the Ukraine conflict from the occupied territories are not available. In the future it might be possible to use technology for collecting real time data in territories occupied. However, these data can only be obtained if data protection and safety of the study participants is possible. We acknowledge that new wars, such as the war in Ukraine and in Sudan, are happening at the moment. The current review aimed to synthesize data from the time period January1994 – June 2022. As it is difficult to keep up to date as new wars are emerging almost on a regular basis we acknowledge that our review might provide knowledge for a certain time period (January 1994 – October 2024) and needs a regular update. Further approaches such as a living systematic review may build on our review. This living review could evaluate the association of human rights violations and mental health conditions and integrate continuously data on emerging human rights violations. One limitation of the review is that we did not include grey literature which could include further groups of forcibly displaced persons. Further research could involve conducting a systematic search of the grey literature. Grey literature could help incorporate information about additional groups of forcibly displaced individuals.

Additionally, we conducted subgroup analyses to investigate potential sources of heterogeneity. Subgroups were combined when the number of studies in each subgroup category was less than four which would have affected the pooled prevalence rates and effect sizes in corresponding analysis. Also, some subgroup analyses were not feasible due to a lack of data availability. Furthermore, our review might have missed some relevant studies. Nevertheless, our review provides the first empirical evidence on the association between human rights violations and mental health conditions. Future research should expand the scope of assessment to include detailed information on the nature, duration, and type of human rights violations. Additionally, further studies are needed to interpret the pathways through which human rights violations impact mental health and to address the existing knowledge gaps in this area.

## Conclusion

To summarize, the results of this study add to the knowledge of mental health conditions of FDPs. The findings highlight the association between human rights violations and mental health conditions among forcibly displaced populations. The study results are relevant for other conflict-affected and persecuted communities where basic human rights are systematically violated. It might be of value to conduct in the future a scoping review on this topic including grey literature. While mental health services are crucial for addressing anxiety, depression, and PTSD, they alone cannot fully alleviate the burden. To make an impact, policymakers, politicians, and service providers must continue to provide mental health services aimed at reducing the mental health conditions among these population groups. However, addressing mental health conditions alone is not sufficient to reduce the burden of mental health conditions among these populations. Further research is required to study the effects of systematic continuous human rights violations in conflict prone areas is essential to identify methods to alleviate the burden of these mental health conditions in FDPs. This effort will require substantial resources and long-term advocacy by all major stakeholders involved in this area.

## Data Availability

The datasets presented in this article are not readily available because datasets are available on request from the corresponding author. Requests to access the datasets should be directed to Jutta Lindert, jutta.lindert@hs-emden-leer.de.
