# Supplementary material for: Human rights violations are associated with forcibly displaced population’s mental health—a systematic review and meta-analysis
Source: Front Public Health. 2025 Jan 16;12:1454331. doi: 10.3389/fpubh.2024.1454331 (PMC11789535; doi:10.3389/fpubh.2024.1454331)

**Supplementary material:**

**Supplementary Table 1:** Search strategy for PubMed, EMBASE, PsycINFO (EBSCO), PTSDPubs (ProQuest), Web of Science

| PubMed (NCBI) | ("Transients and Migrants"[Mesh] OR "Refugees"[Mesh] OR migrant*[tiab] OR unaccompanied minor*[tiab] OR unaccompanied child*[tiab] OR refugee*[tiab] OR displaced person*[tiab] OR displaced people*[tiab] OR forcibly displac*[tiab] OR asylum[tiab])  AND  ("Depressive Disorder"[mesh] OR "Depression"[tiab] OR "Anxiety Disorders"[Mesh] OR depression[tiab] OR depressive[tiab] OR anxiety[tiab] OR panic[tiab] OR obsessive compulsive[tiab] OR ocd[tiab] OR "Trauma and Stressor Related Disorders"[Mesh] OR stress disorder*[tiab] OR stress symptom*[tiab] OR stress syndrome*[tiab] OR traumatic stress*[tiab] OR posttraumatic stress*[tiab] OR traumatic psycho*[tiab] OR posttraumatic psycho*[tiab] OR traumatic neurosis[tiab] OR ptsd[tiab] OR "Anger"[Mesh] OR "Hostility"[Mesh] OR anger[tiab] OR rage[tiab] OR hostility[tiab] OR "Substance-Related Disorders"[Mesh] OR substance abuse[tiab] OR drug use*[tiab] OR drug abuse*[tiab] OR alcohol*[tiab] OR alcoholic*[tiab] OR drinking[tiab] OR opioid*[tiab] OR opiate*[tiab] OR narcotic*[tiab] OR heroin[tiab] OR morphine[tiab] OR opium[tiab] OR amphetamine*[tiab] OR glue[tiab] OR inhalant*[tiab])  AND  ("2009/01/01"[PDAT] : "3000/12/31"[PDAT]) |
| --- | --- |
| Embase (Elsevier, 1974-) | 1. ('refugee'/exp OR migrant*:ab,ti OR (unaccompanied NEXT/1 (minor* OR child*)):ab,ti OR refugee*:ab,ti OR (displaced NEXT/1 (person* OR people)):ab,ti OR 'forcibly displaced':ab,ti OR asylum:ab,ti) 2. ('depression'/exp OR 'anxiety disorder'/exp OR 'anger'/exp OR 'hostility'/exp OR 'addiction'/exp OR depression:ab,ti OR depressive:ab,ti OR anxiety:ab,ti OR panic:ab,ti OR 'obsessive compulsive':ab,ti OR ocd:ab,ti OR (stress NEXT/1 (disorder* OR symptom* OR syndrome*)):ab,ti OR ((traumatic OR posttraumatic) NEXT/1 (stress* OR psycho* OR neurosis)):ab,ti OR ptsd:ab,ti OR anger:ab,ti OR rage:ab,ti OR hostility:ab,ti OR 'substance abuse':ab,ti OR (drug NEXT/1 (use* OR abuse*)):ab,ti OR alcohol*:ab,ti OR drinking:ab,ti OR opioid*:ab,ti OR opiate*:ab,ti OR narcotic*:ab,ti OR heroin:ab,ti OR morphine:ab,ti OR opium:ab,ti OR amphetamine*:ab,ti OR glue:ab,ti OR inhalant*:ab,ti) 3. #1 AND #2 AND [2009-2016]/py |
| PsycINFO (EBSCO)  Limit to 2009 to and later publication date | (DE ("Refugees" OR "Asylum Seeking") OR TI ((unaccompanied W1 (minor* OR child*)) OR refugee* OR (displaced W1 (person* OR people)) OR "forcibly displaced" OR asylum) OR AB ((unaccompanied W1 (minor* OR child*)) OR refugee* OR (displaced W1 (person* OR people)) OR "forcibly displaced" OR asylum))  AND  DE ("Major Depression" OR "Dysthymic Disorder" OR "Reactive Depression" OR "Recurrent Depression" OR "Treatment Resistant Depression" OR "Anxiety Disorders" OR "Acute Stress Disorder" OR "Death Anxiety" OR "Generalized Anxiety Disorder" OR "Obsessive Compulsive Disorder" OR "Panic Disorder" OR "Phobias" OR "Post-Traumatic Stress" OR "Posttraumatic Stress Disorder" OR "Separation Anxiety Disorder" OR "Anger" OR "Hostility" OR "Addiction" OR "Alcoholism" OR "Drug Addiction" OR "Drug Dependency" OR "Heroin Addiction" OR "Drug Abuse" OR "Alcohol Abuse" OR "Drug Dependency" OR "Inhalant Abuse" OR "Polydrug Abuse") OR TI (depression OR depressive OR anxiety OR panic OR "obsessive compulsive" OR ocd OR (stress W1 (disorder* OR symptom* OR syndrome*)) OR ((traumatic OR posttraumatic) W1 (stress* OR psycho* OR neurosis)) OR ptsd OR anger OR rage OR hostility OR "substance abuse" OR (drug W1 (use* OR abuse*)) OR alcohol* OR drinking OR opioid* OR opiate* OR narcotic* OR heroin OR morphine OR opium OR amphetamine* OR glue OR inhalant*) OR AB (depression OR depressive OR anxiety OR panic OR "obsessive compulsive" OR ocd OR (stress W1 (disorder* OR symptom* OR syndrome*)) OR ((traumatic OR posttraumatic) W1 (stress* OR psycho* OR neurosis)) OR ptsd OR anger OR rage OR hostility OR "substance abuse" OR (drug W1 (use* OR abuse*)) OR alcohol* OR drinking OR opioid* OR opiate* OR narcotic* OR heroin OR morphine OR opium OR amphetamine* OR glue OR inhalant*) |
| PTSDPubs (ProQuest) | SU.EXACT("Asylum Seekers" OR "Refugees") OR ti((unaccompanied PRE/1 (minor* OR child*)) OR refugee* OR (displaced PRE/1 (person* OR people)) OR "forcibly displaced" OR asylum) OR ab((unaccompanied PRE/1 (minor* OR child*)) OR refugee* OR (displaced PRE/1 (person* OR people)) OR "forcibly displaced" OR asylum)  limit to 2009-2017 |
| Web of Science  Indexes=SCI-EXPANDED, SSCI, A&HCI Timespan=2009-2022 | TS=(("unaccompanied" NEAR/1 ("minor*" OR "child*")) OR "refugee*" OR ("displaced" NEAR/1 (person* OR people)) OR "forcibly displaced" OR "asylum")  AND  TS=("depression" OR "depressive" OR "anxiety" OR "panic" OR "obsessive compulsive" OR "ocd" OR ("stress" NEAR/1 ("disorder*" OR "symptom*" OR "syndrome*")) OR (("traumatic" OR "posttraumatic") NEAR/1 ("stress*" OR "psycho*" OR "neurosis")) OR "ptsd" OR "anger" OR "rage" OR "hostility" OR "substance abuse" OR ("drug" NEAR/1 ("use*" OR "abuse*")) OR "alcohol*" OR "drinking" OR "opioid*" OR "opiate*" OR "narcotic*" OR "heroin" OR "morphine" OR "opium" OR "amphetamine*" OR "glue" OR "inhalant*") |

**Supplementary Figure 1:** PRISMA Flow diagram

Additional records identified through other sources

(n=611)

Articles identified through electronic data base searching (PubMed, PsychINFO, Web of Science)

(n=7944)

**Identification**

Records after duplicates removed

(n=8555)

**Screening**

Records excluded

(n=8314)

Records screened

(n=8555)

Full text articles excluded with reasons (n=186)

| Age group <18 | (n=14) |
| --- | --- |
| Clinical setting | (n=55) |
| Duplicates | (n=2) |
| Interventional study  No data available | (n=4)  (n=6) |
| No standardized outcome measure | (n=9) |
| Other outcomes | (n=44) |
| Other population | (n=21) |
| Qualitative study | (n=3) |
| Sample size <100 | (n=14) |
| Theories/concepts | (n=14) |

Full text articles assessed for

eligibility

(n=241)

**Eligibility**

Studies included in qualitative synthesis

(n=55)

**Included**

Abbreviation**:** PRISMA: Preferred Reporting Items for Systematic Reviews and Meta-Analyses

**Supplementary Figure 2: Study authors, year, sample size, anxiety prevalence rates with 95% confidence intervals and random % weight by very high versus high risk of bias**


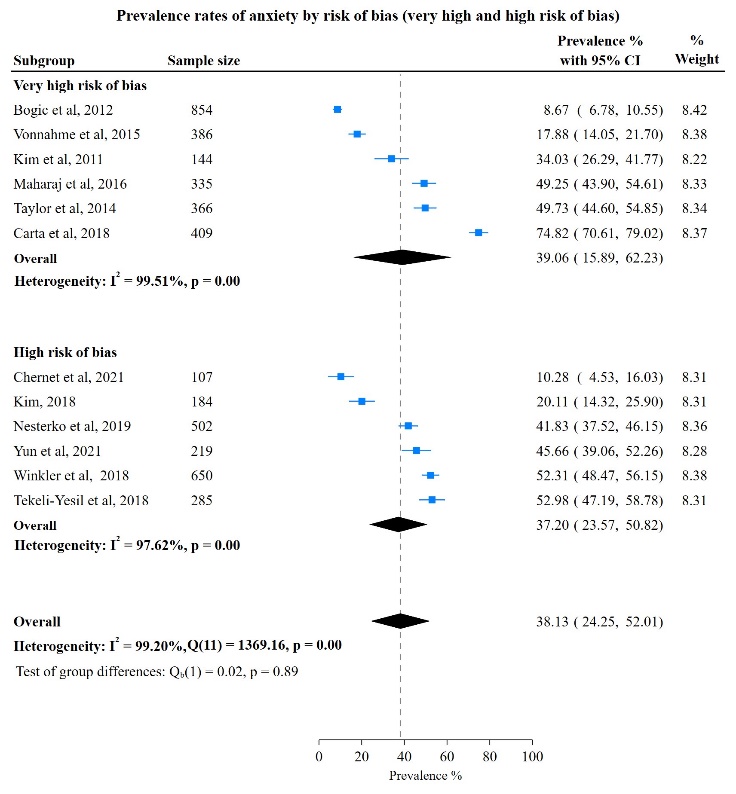


**Supplementary Figure 3: Study authors, year,**

**sample size, anxiety prevalence rates with 95%**

**confidence intervals and random % weight by**

**very high versus moderate, very low risk of bias**


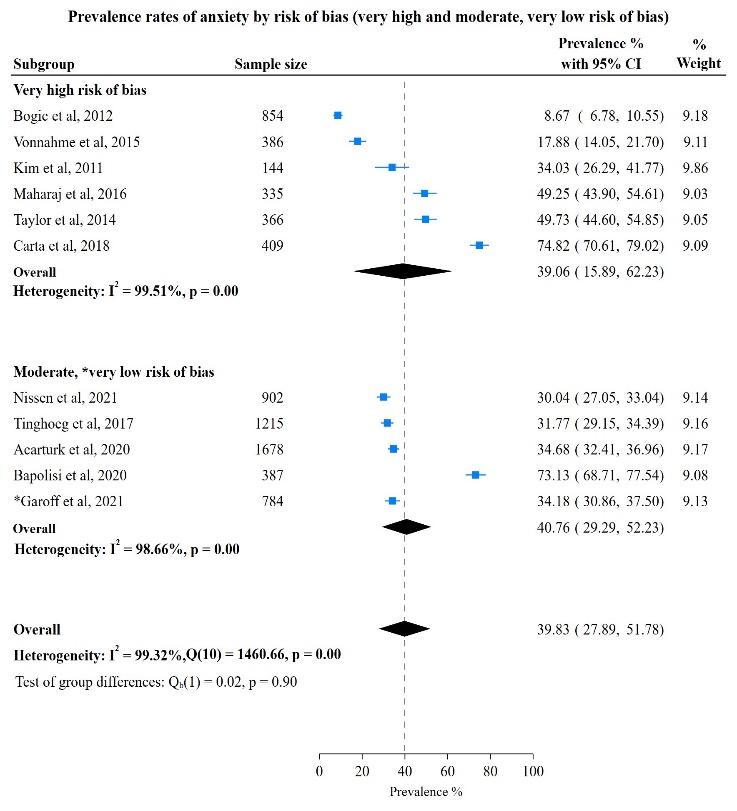


^*^Study with very low risk of bias

**Supplementary Figure 4: Study authors, year,**

**sample size, depression prevalence rates with 95%**

**confidence intervals and random % weight by**

**very high versus high risk of bias**


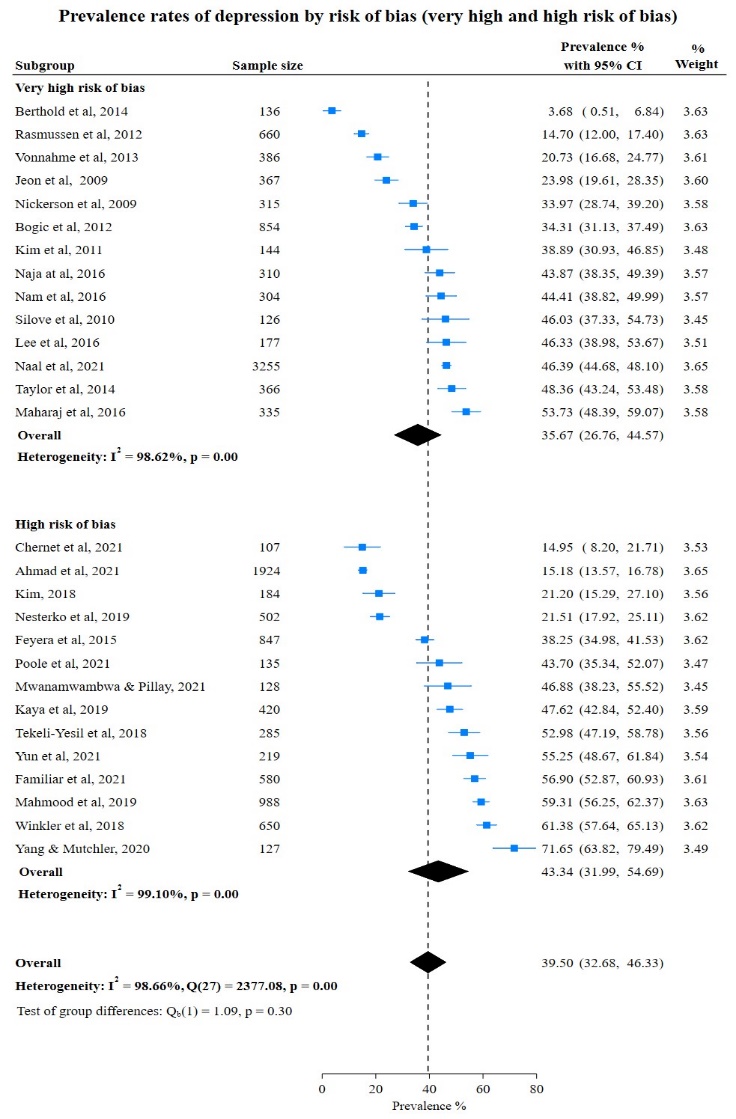


**Supplementary Figure 5: Study authors, year,**

**sample size, depression prevalence rates with 95%**

**confidence intervals and random % weight by very**

**high versus moderate risk of bias**


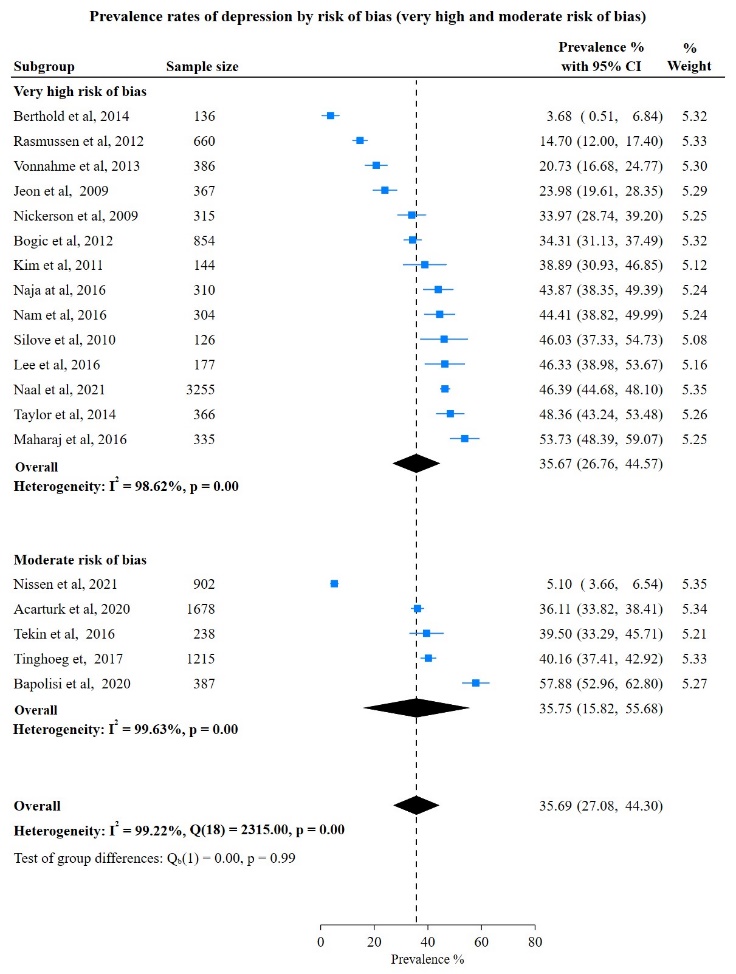


**Supplementary Figure 6: Study authors, year,**

**sample size, depression prevalence rates with 95%**

**confidence intervals and random % weight by very**

**high versus low, very low risk of bias**


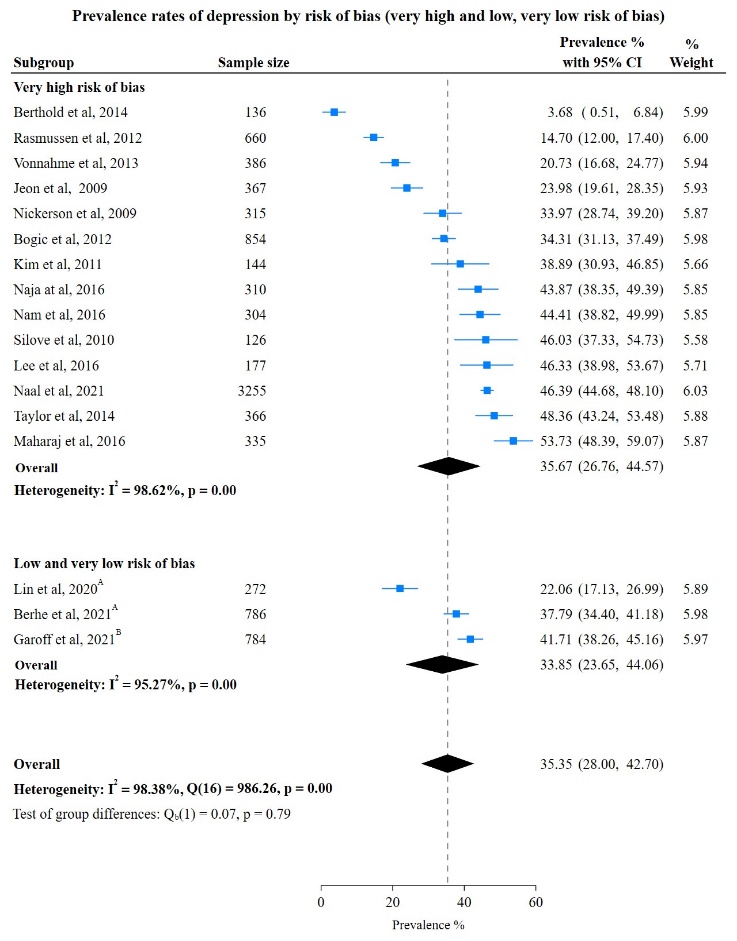


^A^Low risk of bias;

^B^very low risk of bias

**Supplementary Figure 7: Study authors, year,**

**sample size, PTSD prevalence rates with 95%**

**confidence intervals and random % weight by very**

**high versus high risk of bias**


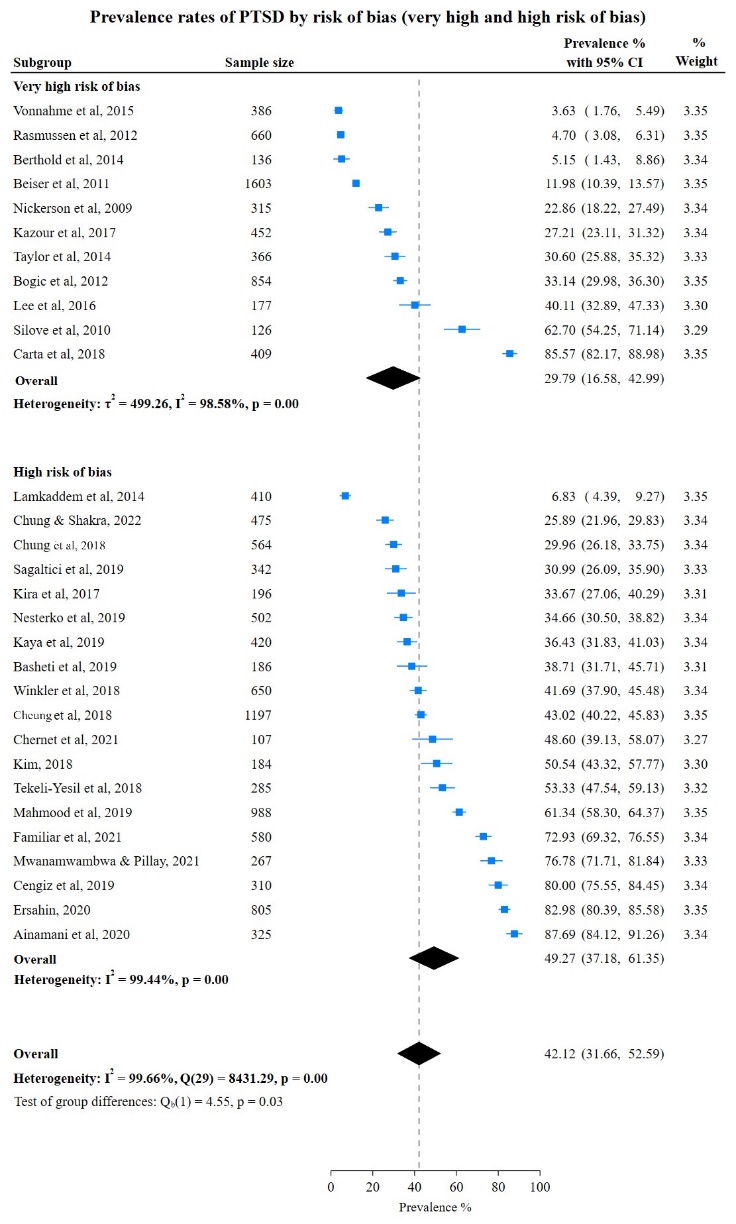


**Supplementary Figure 8: Study authors, year,**

**sample size, PTSD prevalence rates with 95%**

**confidence intervals and random % weight by very**

**high versus moderate, low risk of bias**


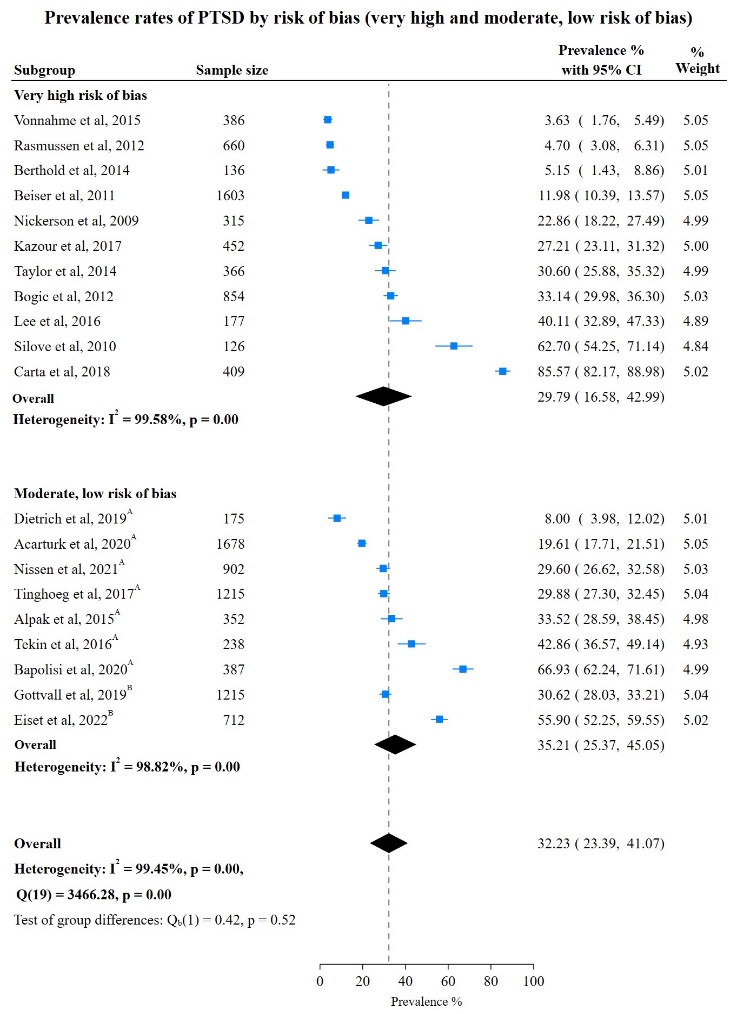


^A^Moderate risk of bias;

^B^Low risk of bias

**Supplementary Figure 9: Study authors, year,**

**sample size, anxiety prevalence rates with 95%**

**confidence intervals and random % weight by**

**convenience versus random sampling**


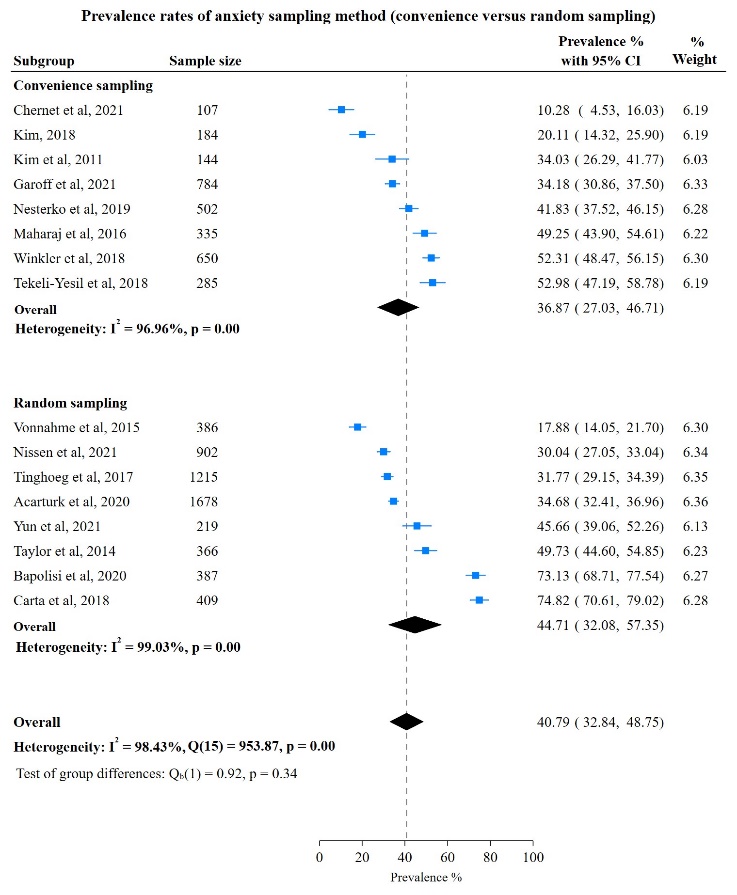


**Supplementary Figure 10: Study authors, year,**

**sample size, depression rates with 95%**

**confidence intervals and random % weight by**

**convenience versus random sampling**


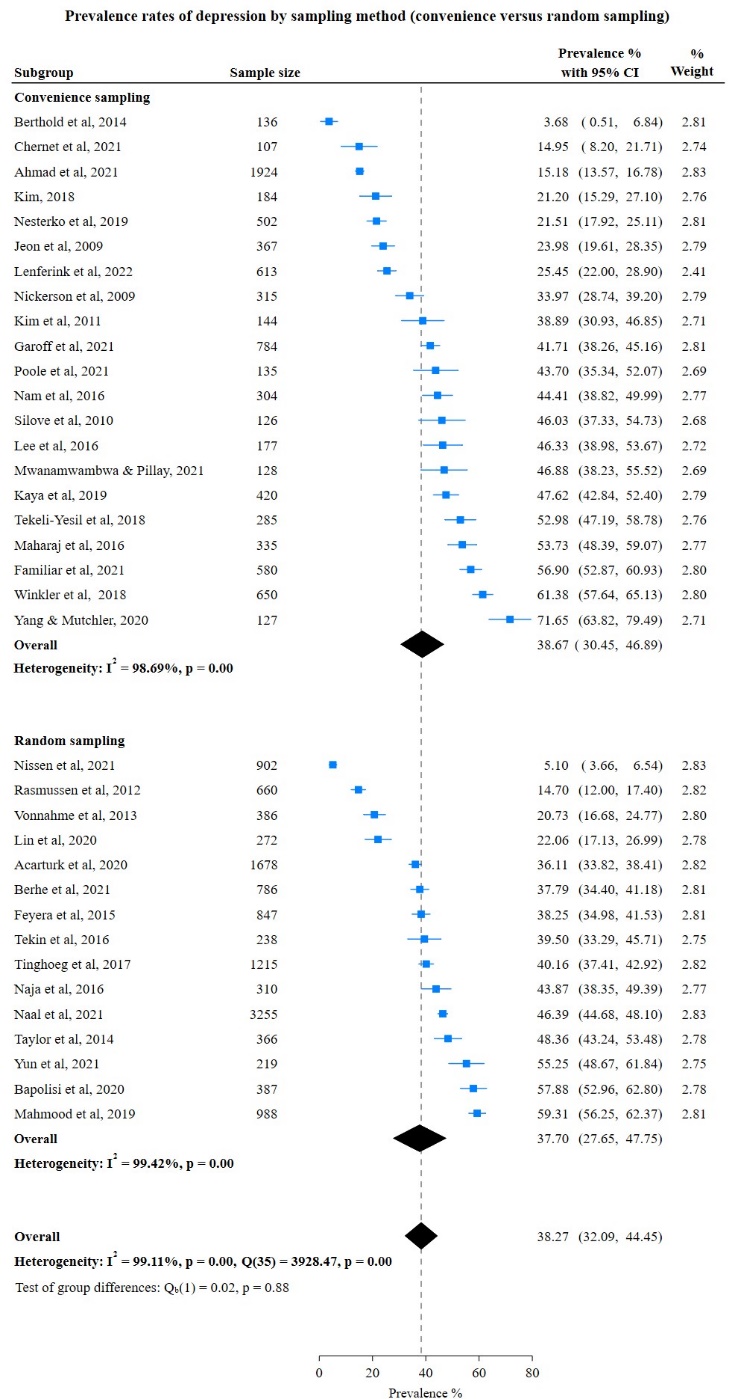


**Supplementary Figure 11: Study authors, year,**

**sample size, PTSD prevalence rates with 95%**

**confidence intervals and random % weight by**

**convenience versus random sampling**


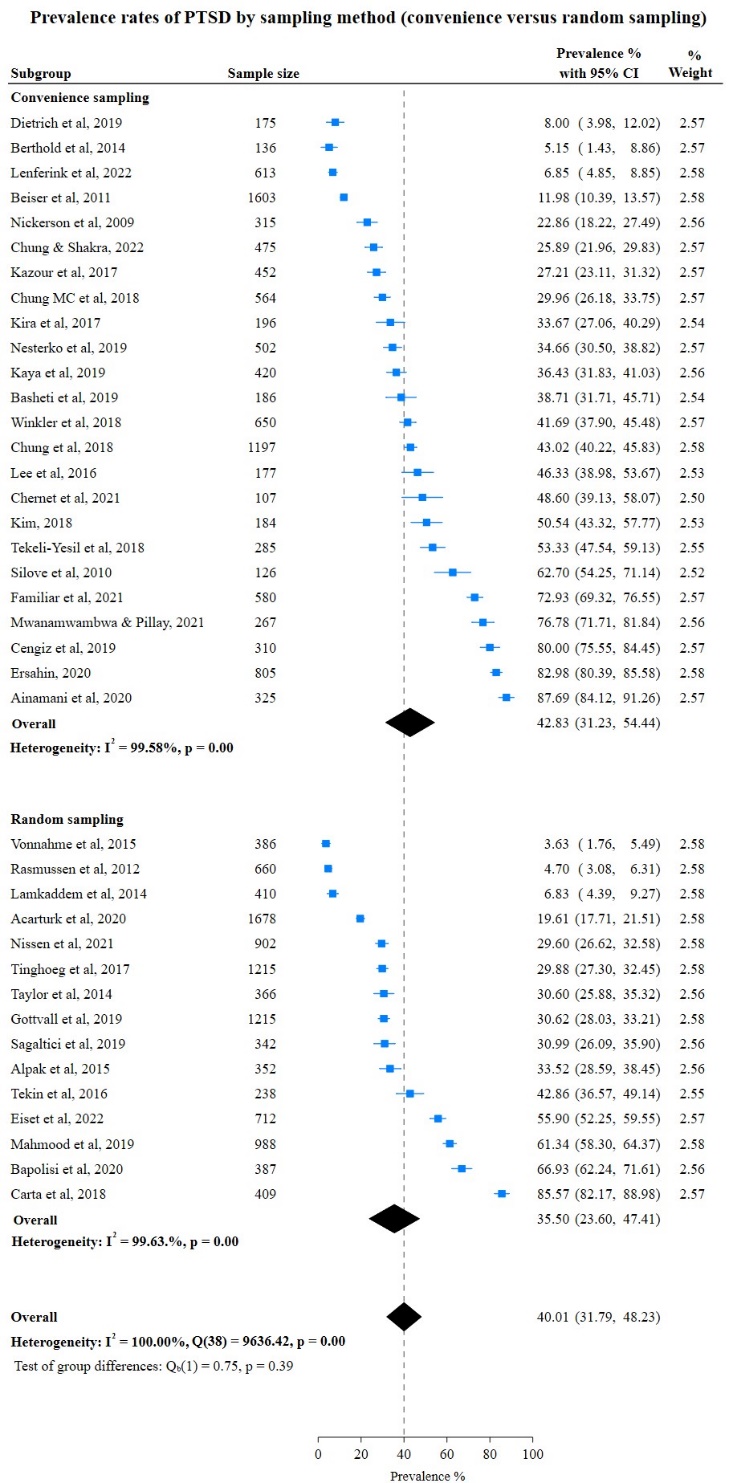

Supplement: Supplementary file 1 [file Data_Sheet_1.docx]
